# Key Features Relevant to Select Antigens and TCR From the MHC-Mismatched Repertoire to Treat Cancer

**DOI:** 10.3389/fimmu.2019.01485

**Published:** 2019-06-28

**Authors:** Stefan Audehm, Manuel Glaser, Matteo Pecoraro, Eva Bräunlein, Sabine Mall, Richard Klar, Manuel Effenberger, Julian Albers, Henrique de Oliveira Bianchi, Janet Peper, Nahid Yusufi, Dirk H. Busch, Stefan Stevanović, Matthias Mann, Iris Antes, Angela M. Krackhardt

**Affiliations:** ^1^Klinik und Poliklinik für Innere Medizin III, Klinikum rechts der Isar, Technische Universität München, Munich, Germany; ^2^Center for Integrated Protein Science at the Department for Biosciences, Technische Universität München, Freising, Germany; ^3^Department of Proteomics and Signal Transduction, Max Planck Institute of Biochemistry, Martinsried, Germany; ^4^Institut für Medizinische Mikrobiologie, Immunologie und Hygiene, Technische Universität München, Munich, Germany; ^5^Eberhard Karls University Tübingen, Interfaculty Institute for Cell Biology, Tübingen, Germany; ^6^Nuklearmedizin, Klinikum rechts der Isar, Technische Universität München, Munich, Germany; ^7^Partner Site Tübingen, German Cancer Consortium (DKTK), Tübingen, Germany; ^8^Partner Site Munich, German Cancer Consortium (DKTK), Munich, Germany; ^9^German Cancer Research Center (DKFZ), Heidelberg, Germany

**Keywords:** T-cell receptor (TCR), peptide-MHC modeling (p-MHC modeling), adoptive T-cell transfer therapy, TCR cell therapy, TCR identification, TCR characterization, trimolecular complex (TCR-p-MHC), target antigen characterization

## Abstract

Adoptive transfer of T cells transgenic for tumor-reactive T-cell receptors (TCR) is an attractive immunotherapeutic approach. However, clinical translation is so far limited due to challenges in the identification of suitable target antigens as well as TCRs that are concurrent safe and efficient. Definition of key characteristics relevant for effective and specific tumor rejection is essential to improve current TCR-based adoptive T-cell immunotherapies. We here characterized in-depth two TCRs derived from the human leukocyte antigen (HLA)-mismatched allogeneic repertoire targeting two different myeloperoxidase (MPO)-derived peptides presented by the same HLA-restriction element side by side comprising state of the art biochemical and cellular *in vitro, in vivo*, and *in silico* experiments. *In vitro* experiments reveal comparable functional avidities, off-rates, and cytotoxic activities for both TCRs. However, we observed differences especially with respect to cytokine secretion and cross-reactivity as well as *in vivo* activity. Biochemical and *in silico* analyses demonstrate different binding qualities of MPO-peptides to the HLA-complex determining TCR qualities. We conclude from our biochemical and *in silico* analyses of peptide-HLA-binding that rigid and high-affinity binding of peptides is one of the most important factors for isolation of TCRs with high specificity and tumor rejection capacity from the MHC-mismatched repertoire. Based on our results, we developed a workflow for selection of such TCRs with high potency and safety profile suitable for clinical translation.

## Introduction

Adoptive T-cell transfer of immune receptor transgenic T cells has demonstrated high clinical potential especially for chimeric antigen receptors (CAR) (1). TCRs, however, are far behind CARs with respect to clinical translation. A number of candidate TCRs have been proposed (2–4), however, only a few clinical trials have been published (5, 6) likely explainable by the challenging selection process for suitable peptide antigens as well as equally potent and safe TCRs.

Diverse qualities of TCRs have been described to be essential and potentially predict anti-tumor reactivity *in vivo* as especially functional avidity and affinity of TCRs (7–11). In addition, slow dissociation half-life of TCRs from peptide-MHC complexes (p-MHC) has been reported to correspond with *in vivo* activity (12). There are less recommendations for high avidity TCRs deriving from the allogeneic or xenogeneic environment or selected by affinity maturation. This is especially important as these TCR may harbor an enhanced risk profile for crossreactivity and therefore toxicity (13, 14). Furthermore, the functionality and efficiency of transferred TCR cell-surface expression depend on the intrinsic quality of the TCR complex (15) and the T-cell specificity might be affected by the formation of mixed heterodimers composed of endogenous and transgenic TCR chains (16).

Suitable target epitope selection remains equally difficult despite the large number of possible human leukocyte antigen (HLA)-peptide ligands. Peptide-HLA binding affinity has been identified as particular important (17, 18). Candidate epitopes are often selected by prediction algorithms. Sequence- and stability-based p-MHC binding predictions are valuable tools (19, 20) to get approximated binding qualities (21) and are used for initial peptide-candidate screenings (22). In addition, combined approaches using sequence- and structure-based algorithms have been applied (23, 24). Peptide ligands to be used as target antigens in cancer can be also directly identified by immunopeptidomics (25, 26) to be potentially used in combination with *in-silico* p-MHC binding prediction (26, 27).

Defining priorities for the selection of epitope and TCR candidates, including the non-self-repertoire, would be an important step to foster clinical translation. We here present an in-depth characterization and comparison of two TCRs identified in a single HLA-mismatched allorestricted approach (sHLAm) recognizing two different peptides derived from myeloperoxidase (MPO) sharing the same restriction element HLA-B^*^07:02 (HLA-B7). One of the TCRs has been previously described as highly specific and tumor-reactive (27, 28). Identification and characterization of the second TCR are described in this manuscript. State of the art key experiments investigating functional qualities of TCR-transgenic TCR as well as in-depth target peptide characterization have been applied to address the question, which set of *in vitro* and *in silico* analyses may support straight forward selection of promising peptide and TCR candidates suitable for clinical translation.

## Materials and Methods

### Cell Lines and T Cells

The isolation of peripheral blood mononuclear cells (PBMC), the isolation of naïve CD8+ T cells and the culturing of target cell lines were realized as described previously (27, 28). For analyses of the MPO-specific TCRs the following cell lines were used: NB-4 (Cell Lines Service, CLS, Germany), SiG-M5 (DSMZ), K562 (ATCC CCL-243), KG1a (CLS), HL-60 (CLS), ML2 (The CABRI consortium), C1R (26), NSO-IL15 (kindly provided by S.R. Riddell), 293Vec-RD114 (BioVec Pharma), and variant types of lymphoblastoid cell lines (LCL) (kindly provided by Steve Marsh). Cell culturing was done as previously described (27). All cell lines were periodically tested for mycoplasma negative status by PCR and cell line authentication was done by flow cytometry-based analyses of cell surface markers and HLA-A^*^- and HLA-B^*^ typing by next-generation sequencing (Center for Human Genetics and Laboratory Diagnostics, Munich, Germany).

### Antibodies and HLA Multimers

Antibodies used for activation of T cells and flow cytometry: anti-hCD3 FITC [UCHT1; Becton Dickinson, Franklin Lakes, NJ, USA (BD)], anti-hCD4 APC/Pacific Blue (RPA-T4; BD), anti-hCD8 APC/V450 (RPA-T8; BD), anti-hCD62L PE, anti-hCD45RO PE (UCHL1; BD), anti-hCD45RA APC (HI100; BD), anti-mouse TCR-β chain (anti-TCRm) FITC/PE/APC (H57-597, BD), anti-hCD45 APC [J.33; Beckman Coulter (BC)], anti-hCD3 AF700 (UCHT1; BD), anti-hCD5 PECyTM5 (UCHT2; BD), anti-hCD4 V450 (RPA-T4; BD), anti-HLA-B7 PE (BB7.1; Merck). HLA multimers were synthesized as previously described (29).

### Selection and Expansion of T Cells Specific for the MPO Ligands

Expansion of T cells specific for the HLA-B7 MPO_2_-ligand was performed as described previously (27). T cells were subsequently screened for functionality. Prior to the multimer sort, expanded T cells from the MPO_2_ single HLA-mismatch setting were co-incubated with lethally irradiated (30 Gy) C1R-B7 tumor cells to remove unspecific HLA-B7-alloreactive T cells (30). After 12 h of co-incubation, the T cells were stained with an anti-human CD137-APC antibody and depleted by MACS sorting (Miltenyi) according to the manufacturer instructions. Subsequently, peptide-specific T cells were sorted using HLA multimers on a flow cytometric cell sorter (MoFlo; Dako, Glostrup, Denmark). HLA-multimer positive T cells were cloned by limited dilution.

### TCR Isolation and Retroviral TCR Transfer

The usage of TCR variable alpha and beta domains were determined by PCR followed by Sanger Sequencing as described previously (31). After *in silico* murinization and cysteine modification of the constant domains of the TCR and insertion of an additional cysteine bridge, the complete sequence was codon-optimized (Genscript, Piscataway Township, NJ, USA) and cloned by a bi-cistronic construct, consisting of both TCR chains into the pMP71 backbone. To produce retroviral supernatants, the embryonal kidney cell line 293Vec-RD114 (BioVec Pharma, Québec, Canada) was used and a retroviral transduction protocol for TCR- and HLA-allele transduction as described previously (32).

### HLA-Vector Constructs Used for T-Cell Clone Isolation, Testing of Specificity, and HLA-Peptide Restriction

The different tumor cell lines without endogenous gene expression of MPO or the desired restriction element HLA-A1, HLA-A2, HLA-B7, HLA-B15, or HLA-B44, respectively, were transduced with the retroviral vector pMP71 containing the HLA of interest attached to green fluorescent protein (GFP) or MPO attached to Discosoma Red Fluorescent Protein (DsRed) Express II. The sequences were cloned into the vector as a bi-cistronic construct separated by the porcine teschovirus-1-derived peptide element P2A. For the generation of *in vitro* transcribed (IVT) mRNA the bi-cistronic constructs were cloned into the plasmid pcDNA3.1(–) (Thermo Fisher Scientific). HLA-allele retroviral transduced cell lines are indicated with the appendix e.g., “-B7”.

### MPO, MPO_2_, and MPO_5_ Mini-Gene Design and Expression in Target Cell Lines

Gene fragments containing the MPO_2_ or MPO_5_ sequence in the center were amplified by designing primers resulting in oligonucleotides of an overall length of ~200 base pairs flanked by a start and a stop codon. The following primers were used for the cloning of MPO_2_- and MPO_5_-mini-gene: 5′-TACAGGCGGCCGCCACCATGACGGCGGTGAGGGC-CGC-3′, 5′-TAGTCGACGGGGCTGCGTCTGTTGTTGC-3′, and 5′TACAGGCGGCCGC-CACCATGCTGGCAGGGGACACCCG-3′, 5′-TAGTCGACGTACTTCCTCATGGCCGTTG3′ using the MPO gene derived from NB-4 cells as template. For the expression of mini-genes in HLA-B^*^07:02-positive target cells, amplified oligonucleotides were cloned into the retroviral vector MP71 containing also dsRed Express II as selection marker. Retroviral supernatant production and retroviral transduction was done as previously described (32).

### Functional Characterization of T-Cell Clones and TCR-Transgenic T Cells

The cytotoxic reactivity of T cells was tested by flow cytometry making use of changes in signal of the HLA-B7eGFP-transgenic target cells during co-cultivation for different time periods and effector to target ratios. For relative quantification of cytotoxicity, *AccuCheck* counting beads were used according to the manufacturer's protocol (Thermo Fisher Scientific) and the results were normalized to either non-TCR-transduced T cells or irrelevant T-cell clones. The supernatants of the co-cultivation were used to measure IFN-γ by ELISA (BD) or GM-CSF, IFN-γ, and lL-2 multiplexed by flow cytometry (Miltenyi Biotec) according to the manufacturer's instructions. The functional avidity of the different TCR was assessed as previously described (27). The 58 HLA-B7 restricted ligands used for the analysis of potential off-target reactivity of TCRF5.4 as tested in co-cultivation experiments, were synthesized (EPS221 synthesizer, Abimed, Langenfeld, Germany) following the 9-fluorenylmethyl-oxycarbonyl/tertbutyl (Fmoc/tBu) methodology. To define peptide residues essential for the recognition by TCRF5.4, amino acid substitution assays using alanine- and threonine variants of the MPO_2_ peptide (Genscript) were performed as described previously (32). Based on the acquired motif, the ScanProsite tool (SIB Swiss Institute of Bioinformatics) was used to identify proteins containing the same pattern. If not stated otherwise, the effector to target ratio was set to 1:1 for the co-incubation assays using 20,000 cells in each fraction.

### UV-Mediated Peptide Exchange

Biotinylated HLA-B7 monomers loaded with the UV-cleavable epitope AARG(J)TLAM were created as previously described (33). Peptide-HLA stability was measured using the protocol on the basis of Rodenko et al. (34). In brief, HLA-B7 monomers loaded with the UV-sensitive peptide were exposed to UV light (366 nm) in the presence of graded amounts (0–200 μM) of MPO peptides. Afterward 1:10 dilutions of the UV-exchanged monomers were adhered via streptavidin to the bottom of a 96-well plate (F96 maxisorp nunc-immune plate, Thermo scientific) and a beta-2-microglobulin ELISA was performed. The OD measurements were performed on a Tecan Sunrise multi-well-reader using Magellan software (version 7.2).

### Flow Cytometry-Based k_off_-Rate Determination of TCRF5.4 and TCR2.5D6

The k_off_-rate was analyzed as described previously (35). In brief, 5 ×10^6^ TCR-transduced T cells were stained with the reversible TCR-specific multimer for 45 min and subsequently incubated with an anti-human CD8 eF450 antibody (Thermo Fisher Scientific) for 20 min. Washed T cells were then stained with 0.2 mg propidium iodide solution for 5 min. 100 μl of the stained TCR-transduced suspension (1–10 ×10^6^ cell/ml) was added to 900 μl FACS buffer prepared in a k_off_-rate FACS tube under constant cool condition (qutools GmbH, Munich, Germany) for the whole measurement. After initiation of analysis in a CyAn ADP Lx 9 color flow cytometer (Beckman Coulter, Miami, US) for 30 s, 1 ml 2 mM D-biotin was injected into the k_off_-rate FACS tube. The measurement was stopped after recording events for a total of 15 min. Data were analyzed using FlowJo v9.5.2 software (FlowJo, LLC, Ashland) and a one-phase exponential decay curve fitting tool of GraphPad Prism (version 7.04, San Diego, USA).

### Human AML Tumor Models and Adoptive T Cell Transfer

The immunocompromised NSG (NOD.Cg-Prkdc^scid^ Il2rg^tm1Wjl^/SzJ) mouse strain was used to establish an NB4 derived myeloid sarcoma model following the experimental procedure as described previously (28). In brief, 1 ×10^7^ NB4-B7eGFP- or NB4-B15eGFP tumor cells were subcutaneously inoculated into the right or left flank, respectively. One day prior to intravenous injection of 2 ×10^7^ TCR-transgenic CD8^+^ T_CM_ (day 8), mice were irradiated with 1 Gy total body irradiation. Irradiated (80 Gy) human interleukin-15 producing murine NSO cells were injected intraperitoneally twice per week (27). The mice were maintained according to conventional institute guidelines and with the approval of local authorities.

### Molecular Modeling

MPO_5_- and the respective variant-HLA-B7 complexes were simulated based on the crystal structure of the HLA-B7 complex bound to the peptide TPQDLNTML (*Protein Data Bank* (PDB) ID: 4U1H) (36) as template. The MPO_2_- and MPO_2_ variant-HLA-B7 complexes were modeled as follows: the MPO_5_-HLA-B7 protein conformations were directly modeled based on 4U1H; for the backbone conformation of the MPO_2_ peptide and its variants the crystal structure of the octamer HLA-B^*^08:01 complex bound to the peptide GGKKKYKL (PDB ID: 1AGD) (37) was taken as no experimental structure of an octamer-HLA-B7 complex was available. For this the HLA α1 and α2 subdomains of 4U1H and 1AGD were structurally aligned using PyMOL, version 1.5.0.4 (Schrödinger). Only the subdomains α1 and α2 of the HLA-B7 protein were considered for modeling. Mutations of the peptide sequences and conformational adaption of the highly flexible binding site residues E70 or R62 were conducted with IRECS (38, 39). Molecular dynamics (MD) simulations and system setup as well as MD analysis were performed with AmberTools16 and the Amber16 software package (40). The Amber ff14SB potential energy function and parameter set (41) were chosen to model the solute. The modeled complexes were simulated in a neutralized (Na^+^, Cl^−^), rectangular box of water molecules [TIP3P force field (42)] with a minimum solute distance of 14 Å to the box boundary. MD simulations were performed with the CUDA compatible GPU version of pmemd, applying periodic boundary conditions (43). Long-range electrostatic interactions were computed with the Particle Mesh Ewald (PME) method (43). A cutoff of 12 Å was used for the computation of non-bonded interactions. MD simulations were performed with a time step of 1 fs. The SHAKE algorithm (44) was applied to constrain bonds involving hydrogen atoms. Temperature and pressure were controlled applying the Berendsen thermostat and barostat (45), using coupling time constants of 0.5 ps (heat up) and 10.0 ps (simulations at 300 K), respectively, and a pressure relaxation time of 2.0 ps. Systems were heated up to room temperature in the NVT ensemble with a sequence of heat up MD simulations at stepwise increasing temperatures totaling up to 1.5 ns (0 K: 10 ps; 5 K, 10 K, 20 K, 50 K: 50 ps; 100 K, 200 K, 200 K: 100 ps; 200 K, 300 K: 200 ps; 300 K: 590 ps), using different restraint settings (0 K, 5 K, 10 K, 20 K: 2.39 kcal mol^−1^ Å^−2^ for all solute atoms; 50 K, 100 K, 200 K: 2.39 kcal mol^−1^ Å^−2^ for all backbone heavy atoms; 200 K: 0.24 kcal mol^−1^ Å^−2^ for all backbone heavy atoms; 200 K, 300 K: 0.24 kcal mol^−1^ Å^−2^ for all backbone heavy atoms of the HLA-B7 protein; 300 K: no restraints). The heated systems were equilibrated in the NPT ensemble for 1.5 ns at 300 K and 1 bar. All systems were simulated for 450 ns in total by performing three independent 150 ns MD simulations per system. Trajectory processing and analysis was performed with cpptraj (46). For the latter, MD frames were extracted from the three trajectories every 100 ps and clustered with respect to their conformation, using the average-linkage hierarchical agglomerative clustering approach (47), applying a minimum cluster distance of 0.75 Å and the best-fit coordinate RMSD of the peptide backbone heavy atoms as distance metric. Hydrogen bonds were calculated applying default settings of cpptraj. Peptide-HLA-B7 protein binding affinities were estimated applying the Molecular Mechanics/Generalized Born Surface Area (MM/GBSA) approach (48, 49) with the single trajectory protocol as implemented in MMPBSA.py (50) using default settings [GB model: OBC-II model (51), atomic radii: mbondi2 (51), no contributions from solvated ions, surface tension applied to the solvent-accessible surface area: 0.0072 kcal mol^−1^ Å^−2^ (52)]. Solute entropy contributions were neglected in the binding affinity estimates. For MM/GBSA free energy computations, snapshots from all three MD replica were combined, discarding the snapshots from the first 25 ns of each MD replicon. For the calculation of root mean square fluctuations (RMSF), each 150 ns-long MD replicon was split in six 25 ns bins and Cα-RMSFs were calculated for each bin separately with cpptraj. To yield the final RMSF values for each MD replicon, the single RMSF values of each bin were averaged. Electrostatic potentials were calculated using the PDB2PQR server (Version 2.0.0) and APBS (Version 1.3) (53, 54), keeping default settings. Figures of peptide-HLA-B7 complexes were generated with VMD (Version 1.9.2) (55).

### Statistics

Statistical analyses of T-cell experiments were performed using GraphPad Prism software version 7.04. Results are presented as standard deviations (SD) of the mean. Samples were compared using the non-parametric Mann-Whitney's *t*-test and multiple *t*-test as indicated in the figure legends.

## Results

### Identification of a Novel HLA-B7 Allorestricted TCR Targeting a New MPO-Derived Epitope

We have previously identified a leukemia-associated HLA-B0702-restricted epitope derived from MPO (MPO_466−474_ named MPO_5_) by mass spectrometry (MS) as well as the TCR2.5D6 derived from the sHLAm T-cell repertoire with high peptide specificity and leukemic reactivity suitable for clinical application in the context of allogeneic stem cell transplantation (27). In our MS-based approach additional MPO epitopes presented on myeloid tumor cells have been identified (27) and selected for stimulation in sHLAm approaches to identify new MPO-specific TCRs. In fact, one novel TCR candidate (TCRF5.4) directed against the MPO_145−152_-peptide (MPO_2_, Table 1) with promising characteristics in early screening analyses was discovered. Shortly summarized, this TCR was identified after prior CD137 depletion of alloreactive T cells as previously described (30) followed by MPO_2_-multimer sorting and T-cell cloning (Supplementary Figures 1A,B). Specific T-cell clones were detected (Supplementary Figure 1C) expressing all the same TCR chains (Vα2 and Vβ1, TCRF5.4). CD8^+^ central memory T cells (T_CM_) transduced with an optimized and cysteine-modified TCRF5.4 construct (59, 60) displayed high transduction efficacy and TCR expression (Supplementary Figure 1D). TCRF5.4-transduced CD8^+^ T_CM_ were highly specific for target cells either pulsed with or endogenously presenting the respective peptide after mini-gene transfer (Supplementary Figure 1E). Moreover, primary malignant cells derived from patients with myeloproliferative neoplasia (MPN) expressing MPO and HLA-B7 were well recognized (Supplementary Figure 1F). To exclude major cross-reactivity, we screened common HLA alleles expressed on different lymphoblastoid cell lines (LCL) pulsed with MPO_2_ and compared the reactivity to non-pulsed conditions (Supplementary Figure 1G; Supplementary Table 1). TCRF5.4-transgenic T cells showed alloreactivity against LCL8 carrying the HLA-alleles A^*^02:04/B^*^51:01. Co-incubation of TCRF5.4-transgenic T cells with C1R-A2 or Tapasin-1 deficient T2 (HLA-A2^+^/HLA-B51^+^) confirmed MPO_2_-independent HLA-B51 cross-reactivity (Supplementary Figure 1H; Supplementary Table 1). However, no recognition of TCRF5.4-transgenic T cells was seen against a set of 58 HLA-B7 restricted peptides (Supplementary Figure 1I). The TCRF5.4 peptide crossreactivity risk was tested on HLA-B7^+^ LCL1 cells by using alanine or threonine substitution variants (Ala/Thr-scan) of MPO_2_ (Supplementary Figures 1J,K). Interestingly, we observed different TCR-binding motifs for both amino acids substitution variants indicating a flexible binding pattern of the TCR toward the p-MHC. Applying the ScanProsite tool (61) for the combined Ala/Thr-scan motif (X-P-A-Q-X-X-V-X) revealed 297 hits outside the MPO source protein whereas only 1 hit was found if the motifs were analyzed separately.

**Table 1:** Characteristics of analyzed p-MHC complexes.

**Peptide numbering derived from Isoform H7 [UniProtKB—P05164-3 (PERM_HUMAN)]; HLA-Restriction: HLA-B7**				**NetMHC 4.0 (22, 56)**	**Syfpeithi (57)**	**NetMHCstab 1.0 (20)**	**Stability after UV-peptide exchange**	**Thermal stability**	
**Alias**	**Peptide**	**Source**	**Sequence**	**Affinity (nM)**	**Score**	**Stability (hrs)**	V_**50**_ (log_**10**_ M)^*^	**V_**50**_ (^**°**^C)**	**Half-life (hrs) 37^**°**^C**
MPO_5_	MPO466	(27)	NPRWDGERL	36.75	23	2.69	−5.535	46.4	4.1
MPO_2_	MPO145	(27)	TPAQLNVL	305.31	n.a.	4.01	−4.671	46.3	3.5
MPO_2_+L1	MPO144		LTPAQLNVL	15292.21	12	0.35	−3.728	44.0	2.9
pp65	417-426	(58)	TPRVTGGGAM	3.86	19	6.04	n.a.	50.0	5.6

* *pooled values of at least three independent experiments, n.a., not available*.

### Differences in Transgenic TCR Expression Between TCRF5.4 and TCR2.5D6 as Well as Quantitative and Qualitative Cytokine Secretion *in vitro*

Based on the qualities of TCRF5.4, the same source protein of recognized peptides and the same restriction element, we decided to compare this TCR to the recently characterized TCR2.5D6 to investigate which TCR may be superior and to define prospective criteria for rapid selection of most suitable allorestricted TCRs for adoptive transfer of TCR-transgenic T cells.

**Figure 1:**
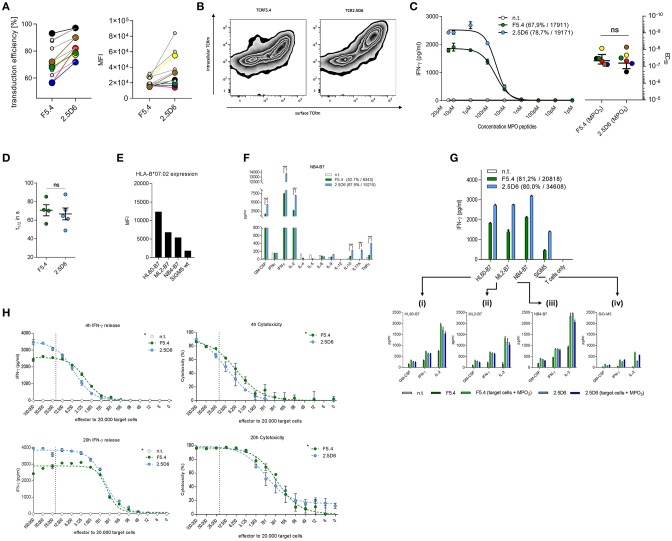
TCR surface expression and quality of cytokine release differs between TCRF5.4- and TCR2.5D6-transduced T cells. **(A)** Transduction efficiency measured by flow cytometry analysis of surface expression (left) and MFI (right) of TCRF5.4 (green) and TCR2.5D6 (blue). Connections between data points symbolize couples of the same recipient T cells used for TCR transduction (*n* = 11). Colored and bigger data points represent pairs of T cells that were also used for the functional avidity analyzes in **(C)**. **(B)** Surface and intracellular anti-TCRm staining of both TCR (X-axis: surface TCRm signal, Y-axis: intracellular TCRm signal) measured by flow cytometry (*n* = 2). **(C)** Functional avidity of TCRF5.4- or TCR2.5D6-transduced PBMC analyzed in response to KG1a-B7 pulsed with graded amounts of MPO_2_ or MPO_5_ at an E/T ratio of 1:1 after 20 h of co-incubation. Half-maximal IFN-γ release was calculated using logarithmic dose-response fitting algorithm with variable slope (EC_50_) of GraphPad Prism. Mean ± SD of triplicates of one representative experiment are shown (*n* = 6). Colored data pairs represent T cell pairs also used in **(A)**. **(D)** Flow cytometry based k_off_-rate measurements of TCRF5.4 (*n* = 4) and TCR2.5D6 (*n* = 5). The dissociation half-lives calculated by one-phase decay algorithm using GraphPad Prism are shown for both TCR. **(E)** MFI of HLA-B7 expression of selected AML cell lines measured by flow cytometry. **(F)** Multi-cytokine release of TCRF5.4 or TCR2.5D6 transduced PBMC in response to the HLA-B7-transgenic AML cell line NB4. NB4 is representatively shown for all 4 tested AML cell lines HL60, ML2, and SiG-M5. Standard deviations of the mean of triplicates are shown (*n* = 2 for each cell line) **(G)** IFN-γ secretion by TCRF5.4 or TCR2.5D6 transduced CD8^+^ T_CM_ in response to AML-cell lines with endogenous MPO expression measured by ELISA. 10.000 effector cells were used in a ratio of 1:1. Standard deviations of the mean of triplicates are shown (*n* = 4). **(G**_**i−iv**_**)** Cytokine secretion (GM-CSF, IFN-γ, and IL-2) measured by flow cytometry based multiplex analysis in response to selected AML cell lines is shown in triplicates either unpulsed or additionally pulsed with MPO_2_ or MPO_5_. **(H)** IFN-γ release (left panels) and cytotoxicity (right panels) of TCRF5.4- or TCR2.5D6-transduced CD8^+^ T_CM_ against NB4-B7 cells analyzed for E/T titrations ranging from 5:1 to 0.0031:1 using a constant target cell amount of 20.000 for different periods of co-cultivation (4 and 20 h). The dashed line in each graph represents theoretically an E/T of 1:1. The percentage of killing was calculated using absolute counts of remaining NB4-B7 target cells normalized to non-transduced T cells by flow cytometry (*n* = 2, ^*^81.2% transduction efficiency for TCRF5.4, 80.0% for TCR2.5D6, MFI TCRF5.4 = 20818, TCR2.5D6 = 34608). **(C,G,H)** Non-transduced T_CM_ were used as negative controls and standard deviations of the mean of triplicates are shown if not otherwise stated. Transduction efficiency and MFI of both TCRs are either bracketed or referred by an asterisk (*) to the legend. IFN-γ ELISA was performed using supernatants of co-incubations **(C,G,H)** and multiplexed cytokine analysis by flow cytometry **(F,G**_**i−iv**_**)**. **(D)** Mann–Whitney test: ns: not significant (*p* > 0.05), **(F)** Multiple *t*-test with false discovery rate (FDR) of 1%, ***p < 0.001.

The TCR-transduction rates of recipient T cells differed in most but not all experiments approximately by 10% as the mean was 72.2% for TCRF5.4 and 82.3% for TCR2.5D6 (Figure 1A, left). The mean fluorescence intensity (MFI) of TCRF5.4 expression was also in many experiments inferior compared to TCR2.5D6 (Figure 1A, right). We observed that TCR2.5D6 is more efficiently expressed as nearly all positive TCR-signal is located on the surface of the T cells whereas TCRF5.4 is not fully expressed (Figure 1B).

Subsequently we investigated the functional avidity of both TCRs toward p-MHC complexes on MPO-negative, HLA-B7-transgenic KG-1a cells. No significant difference in EC_50_ values was observed between both TCR-transduced T cells (Figure 1C, left). The colored data points in Figures 1A,C symbolize the same pairs of transgenic T cells of different TCR-transductions used for the measurements. Our data show that the EC_50_ values were comparable for both TCR independent of the differences in TCR expression. Similarly, k_off_-rate experiments analyzing the dissociation time of both TCRs from the p-MHC complex were also comparable for both TCR (Figure 1D). Thus, functional avidity and k_off_-rate analyses revealed similar results despite differences of TCR-expression and surface density levels.

For the following characterization of both TCRs in co-incubation assays, we used MPO high expressing AML cell lines HL60, ML2, and NB4 (transgenic for HLA-B7) and SiG-M5 (endogenous HLA-B7 expression). HLA-B7 surface expression analysis revealed different levels of HLA-B7 within these cell lines (Figure 1E). First, we used the AML cell lines to investigate an extended cytokine profile of TCRF5.4- and TCR2.5D6-transduced PBMCs. Here we observed differences in the quantity but also quality of cytokine secretion between both TCRs (Figure 1F). The results, representatively shown for NB4, demonstrate increased amounts of IFN-γ, GM-CSF, and IL-2 for TCR2.5D6-transduced PBMCs as well as significantly enhanced secretion of IL-10, IL-17A, and TNFα compared to PBMCs transduced with TCRF5.4. Focusing on CD8^+^ T cells in subsequent assays, all four tumor cell lines with endogenous MPO expression were recognized consistently by TCRF5.4- and TCR2.5D6-transduced T cells, respectively (Figure 1G). Thereby, TCRF5.4-transduced CD8^+^ T cells released substantially lower amounts of IFN-γ, GM-CSF, and IL-2 compared to TCR2.5D6-transduced CD8^+^ T cells in experiments with equal TCR transduction rates but again reduced TCR expression levels. However, these differences could be compensated by additional MPO_2_ peptide pulsing (Figure 1G_i−iv_). In contrast, pulsing of three out of four cell lines with peptide MPO_5_ did not have a major impact on cytokine secretion by TCR2.5D6-transgenic T cells. Our data indicate that HL60, ML2, and NB4 displayed saturating conditions of endogenously expressed MPO_5_ eliciting maximal reactivity of TCR2.5D6 while that was not the case for MPO_2_ and TCRF5.4. In case of SiG-M5 with lowest HLA-B7 expression, cytokine secretion was increased for both MPO-peptides after additional peptide-pulsing.

In subsequent analyses we investigated the impact of effector to target ratio titrations (E/T) on IFN-γ release and cytotoxic capacity of T cells transgenic for both TCR. Again, we observed differences at high E/T ratios with respect to quantity of IFN-γ release (Figure 1H, left panels). However, only minor differences were seen for cytotoxicity of both TCR-transduced T cells after 4 and 20 h of co-incubation (Figure 1H, right panels). At lower E/T ratios the IFN-γ secretion was comparable for both TCR and estimated half maximal E/T ratios for IFN-γ secretion as well as cytotoxicity were slightly favorable for TCRF5.4 at both time points.

### TCR2.5D6 Shows Superior Tumor Control *in vivo* Compared to TCRF5.4

Subsequently, we tested both TCRs using the NB4-tumor model in immune deficient NOD.Cg-Prkdcscid Il2rgtm1Wjl/SzJ (NSG) mice (28) to assess whether TCRF5.4 could compete with TCR2.5D6 *in vivo*.

For initial *in vivo* experiments, we used TCR-transgenic CD8^+^ T_CM_ and traced the growth of subcutaneous established tumors in mice that received either TCRF5.4-, TCR2.5D6- or not transduced T cells. The immunogenicity of the T-cell products was confirmed by functional *in vitro* tests prior to intravenous T-cell injection 8 days after tumor inoculation. The mice were sacrificed 7 days later for *ex vivo* analyses. Both TCR show highly comparable rejection capacity of NB4-B7^+^ tumors in comparison to control tumors (Supplementary Figure 2A) also reflected in significantly reduced tumor weights compared to the non-transduced T cell group (Supplementary Figure 2B). The percentage of CD3^+^ T cells found *ex vivo* within NB4-B7^+^ tumors was similar and significantly enhanced compared to the non-transduced T-cell group and NB4-B15^+^ control tumors (Supplementary Figure 2C). Investigation of T-cell distribution revealed slightly enhanced presence of TCRF5.4-T cells in blood and lung compared to TCR2.5D6-T cells (Supplementary Figure 2D).

**Figure 2:**
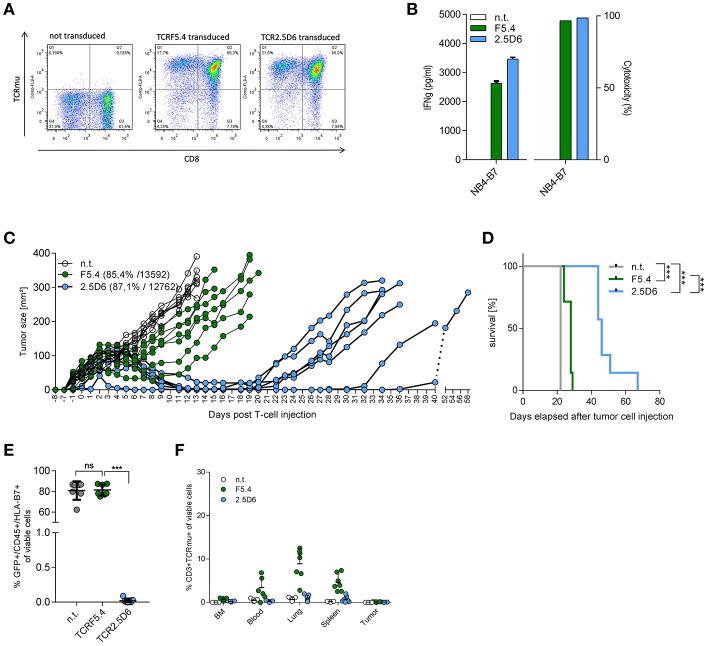
TCR2.5D6-transduced T cells show superior *in vivo* tumor killing efficacy. **(A)** FACS analysis of transduction efficacy for TCRF5.4- and TCR2.5D6-transduced T cells used for the long-term mouse experiment. The plots show pre-gated living CD3^+^ cells. X-axis represents the CD8 marker and Y-axis represents the TCRm^+^ T-cells. **(B)** Functionality of TCR-transduced T cells used for the long-term survival experiment of mice shown in **(C)** measured by IFN-γ release (right) and cytotoxicity (left) after co-cultivation for 24 h with NB4-B7 tumor cells in an E/T ratio of 1:1 *in vitro*. **(C)** Growth kinetic of NB4-B7eGFP tumors in NSG mice for a period of 58 days. Each line represents an individual tumor growth curve per mouse: n.t (*n* = 7), TCRF5.4 (*n* = 7), TCR2.5D6 (*n* = 7). **(D)** Kaplan Meier curve of mice shown in **(C)**, ***p ≤ 0.0002. **(E)** Percentage of GFP^+^/HLA-B7^+^/NB4-B7eGFP tumors cell analyzed *ex vivo* by flow cytometry at individual time points after decease of mice shown in **(C)**. Non-transduced (*n* = 7), TCRF5.4-transduced (*n* = 7) or TCR2.5D6-transduced T cells (*n* = 7), ^ns^*p* ≥ 0.05, ***p = 0.0006. **(F)** Percentage of CD3^+^/TCRm^+^ living cells in BM, blood, lung, spleen, and tumor analyzed *ex vivo* by flow cytometry at individual time points after T-cell injection after decease of mice shown in **(C)**. n.t (*n* = 7), TCRF5.4-transduced (*n* = 7) or TCR2.5D6-transduced T cells (*n* = 7). **(C)** Transduction efficiency and MFI of both TCRs are bracketed. **(E)** Significances are calculated by Mann-Whitney Test. **(D)** Survival statistics are calculated by Log-rank (Mantel-Cox) test.

To assess the long-term survival of mice, we performed transfer experiments with T-cell products that were comparable both in TCR-transduction rate and TCR surface density expression (Figure 2A). Analyzing T cells from this expanded transduction before injection confirmed results from previous experiments with respect to IFNγ secretion and cytotoxicity independent of the equal TCR transduction rate and expression level (Figure 2B). Using our *in vivo* mouse model, we observed a significant improved tumor control (Figure 2C) and median survival (Figure 2D) in TCR2.5D6-treated mice (46 days) compared to TCRF5.4 (28 days). *Ex vivo* analysis of tumors discovered a complete loss of HLA-B7eGFP expression of tumor cells within the TCR2.5D6-group, while the TCRF5.4-group retained the HLA-B7eGFP expression (Figure 2E). We analyzed again the T-cell distribution within each mouse (Figure 2F) and observed only few TCR-transgenic T cells in the BM, blood, lung, spleen or the tumor of the TCR2.5D6 group. In contrast, in the TCRF5.4 group, we detected TCR-transgenic T cells in the blood, lung and the spleen but not within the tumor. These results demonstrate superior tumor control of TCR2.5D6 T cells as well as different forms of tumor escape in both groups.

### Impact of p-MHC Interactions on T-Cell Recognition by Both TCRs

**Figure 3:**
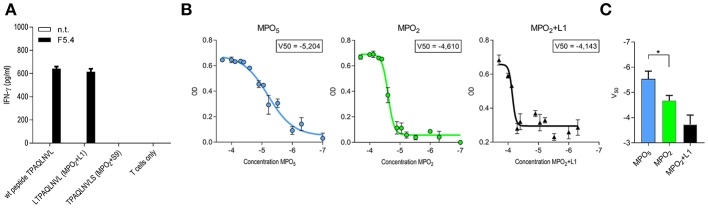
MPO_5_-HLA-B7 complex is superior in comparison to MPO_2_-HLA-B7 with respect to stability and binding quality. **(A)** IFN-γ release by TCRF5.4 together PBMC in response to HLA-B7^+^ LCL1 cell line pulsed with MPO_2_ or the non-americ counter partners LTPAQLNVL (MPO_2_+L1) or TPAQLNVLS (MPO_2_+S9), respectively. An E/T ratio of 1:1 was used. IFN-γ secretion of the supernatants after co-cultivation was measured by IFN-γ ELISA. Standard deviations of the mean of triplicates are shown (*n* = 2). **(B)** Beta-2-microglobulin (B2M)-ELISA of UV-mediated peptide exchanged HLA-B7 monomers. V_50_ values of a Boltzmann fitting for each MPO-peptide was determined by the optical density of B2M after degraded amounts of MPO-peptides were used for HLA-stabilization. Standard deviations of the mean of triplicates are shown (*n* = 3). **(C)** Pooled results of **(B)** for MPO_5_ (*n* = 4), MPO_2_ (*n* = 4), and MPO_2_+L1 (*n* = 3). Significance is calculated by Mann-Whitney Test: *p = 0.0286.

The discrepancy in tumor control observed for both TCR-transduced T-cell groups *in vivo* despite similar functional avidity and k_off_-rate was not simply explainable by differences in TCR expression as this was comparable within the long-term *in vivo* experiment. We therefore focused on peptide MHC (p-MHC) interactions potentially involved in differential function and investigated peptide affinity and HLA-stability of p-MHC binding for both MPO peptides *in vitro* as well as *in silico*. In case of the octamer MPO_2_ we performed additionally pulsing experiments with the N-terminal (LTPAQLNVL = MPO_2_+L1) and C-terminal (TPAQLNVLS = MPO_2_+S9) prolonged variant and detected IFN-γ secretion by TCRF5.4-transduced T cells in response to MPO_2_+L1 but not for MPO_2_+S9 (Figure 3A). Binding predictions of the MPO peptides were applied for either sequence-based (19, 22, 62) or stability-based predictions (20) (Table 1). In sequence-based binding affinity predictions, MPO_2_ was outperformed by MPO_5_. However, considering HLA-complex stability prediction the MPO_2_-MHC complex seemed to be more stable compared to the MPO_5_-MHC complex. Affinity and HLA-stability predictions for MPO_2_+L1 indicated weak performance in both cases.

To verify the stability results of all three MPO-peptides experimentally, we performed HLA-B7 UV-peptide exchange assays with titrated amounts of peptide added to a constant concentration of HLA-B7 monomers bound to a UV-sensitive peptide. Our data showed that MPO_5_ is superior to MPO_2_ with almost one log increased potential to rescue the HLA-B7 molecule (Figures 3B,C; Table 1) while MPO_2_+L1 had the lowest potential for HLA-stabilization. Thus, our results oppose the results of the NetMHC stability prediction. As a second experiment, we performed thermal shift assays for the MPO peptides to characterize their potential to prevent denaturation of the HLA-complex with ascending temperature (Supplementary Figure 3A; Table 1). Interestingly the MPO_2_- and MPO_5_-HLA complex heat-associated denaturation is very similar, as the half maximal melting temperature (V_50_) for both peptides, calculated by Boltzmann fitting, was almost identical (46°C). The MPO_2_+L1 complex is more sensitive to heating (44°C) while the pp65_(417−426)_ peptide, taken as a control, with the highest predicted peptide affinity also has the highest temperature stability (50°C). However, the decay of the MPO HLA-complexes over time at constant 37°C revealed, similar to our UV-exchange and *in silico* sequence-based affinity prediction results, that MPO_5_ had a better stabilizing effect on the MHC complex (4.1 h) compared to MPO_2_ (3.5 h; Supplementary Figure 3B; Table 1). In contrast to stability predictions, we clearly see differences in peptide and HLA-complex dissociation at physiological temperature indicating that MPO_5_ has a longer k_off_-rate compared to MPO_2_.

To investigate whether the peptides are differentially presented on the surface of various target cell lines, we analyzed the surface presentation of both peptides directly on a panel of AML cell lines. We used a targeted MS approach using heavy labeled counterparts of the MPO-peptides as a reference within each sample. In fact, we observed large differences in the detection of both MPO peptides suggesting MPO_2_ to be markedly lower presented on the surface of all AML cells lines compared to MPO_5_ (Supplementary Figure 3C). In addition, there was a similar relative intensity of surface peptide presentation between the different cell lines with SiG-M5 showing the lowest peptide presentation corresponding to the lowest HLA-B7 density and recognition by both TCRs (Supplementary Figure 3C; Figure 1E). However, technical issues need to be considered as MPO_2_ may not be efficiently ionized based on its amino acid sequence. In contrast, the amino acid sequence of MPO_5_, especially the arginines at positions 3 and 8, favor the detection by MS. Thus, comparison of quantitative presentation of both peptides by MS may have limitations.

### Modeling of MPO-Peptide HLA Ligands Confirms Stable HLA Peptide Binding and Reduced Fluctuation of MPO_5_ Within the Binding Cleft Correlating With Reduced Cross-Reactivity

**Figure 4:**
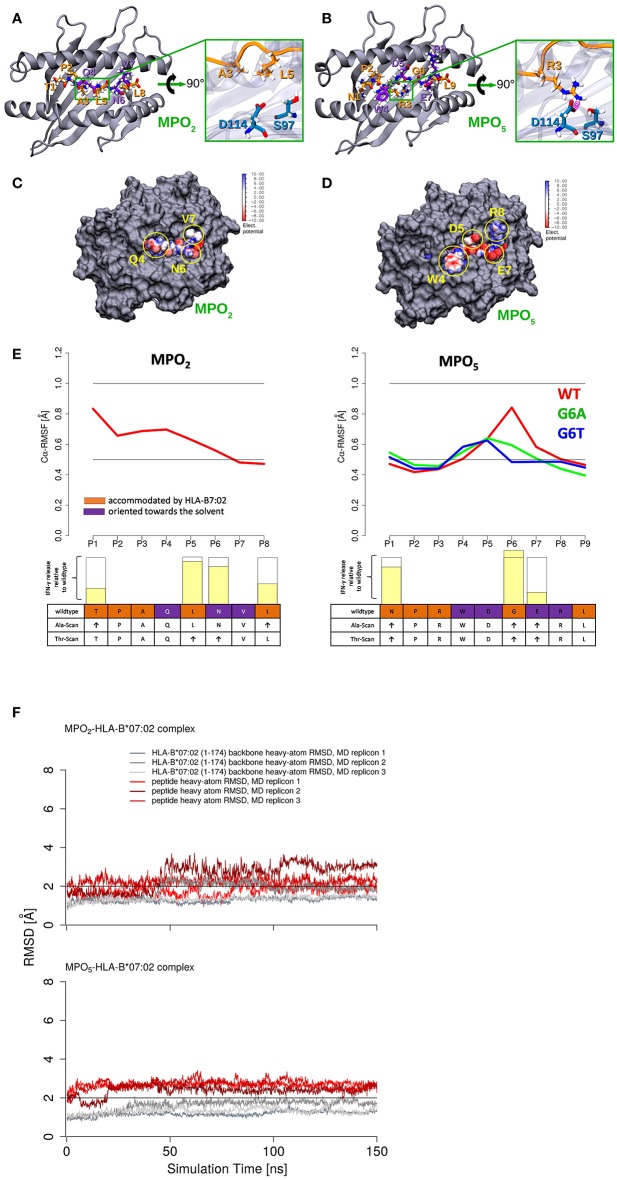
MPO_2/5_-HLA-B7 structure-based modeling revealed less fluctuation of MPO_5_ within the HLA-binding cleft compared to MPO_2_. **(A,B)** Representative conformation of MPO_2/5_ in the bound state. The HLA-B7 protein is shown as cartoon representation (gray), the bound peptides are depicted in stick representation and are highlighted: (hydrogen atoms: white, nitrogen atoms: blue, oxygen atoms: red, carbon atoms: orange or violet), solvent-exposed peptide residues (violet carbon atoms), peptide residues accommodated by the MHC peptide binding cleft (orange carbon atoms). In the green inlays, the HLA-B7 residues S97 and D114 (blue sticks) are shown for a direct comparison of major differences in the MPO_2/5_ HLA-B7 binding modes and anchoring to the HLA-B7 peptide binding cleft. Peptide backbones are shown in orange cartoon representation. **(A)** The green inlay depicts the MPO_2_ residues A3 and L5, accommodated by the MHC peptide binding cleft. **(B)** The green inlay depicts the MPO_5_ residue R3. Hydrogen bonds are highlighted in magenta. **(C,D)** Space filling models showing the TCR interface of the MPO_2/5_-HLA-B7 complexes. Protein is depicted in gray and the bound peptides are depicted as van der Waals (vdW) surface colored according to their electrostatic potential (white: neutral areas, red: negatively charged areas, blue: positively charged areas). Solvent-exposed and TCRF5.4- or TCR2.5D6-accessible residues of the MPO_2/5_-HLA-B7 complexes are highlighted with yellow circles, respectively. **(E)** Calculated root mean square fluctuations (RMSF) of Cα atoms of the HLA-B7-bound MPO_2/5_ peptides (red) and G6A (green)/G6T (blue) variants of MPO_5_ (average of three independent MD simulations). The individual TCR-recognition motif from the alanine- and threonine- (Ala/Thr)-scans is shown together with the IFN-γ release (transparent yellow bars) observed in the Ala- or Thr-scan (Supplementary Figures 1J,K) relative to individual wildtype IFN-γ secretion (empty bars) below the RMSF plots. The height of the empty bars reflects the maximal IFN-γ release elicited by the wildtype peptide, ↑ = interchangeable amino acid residues. The Ala/Thr-scan for MPO_5_ is described elsewhere (27). **(F)** Root mean square deviation (RMSD) of HLA-B7 protein backbone heavy atoms and MPO_2/5_ heavy atoms during MD simulations calculated with respect to the initial energy minimized peptide-HLA-B7 complex models.

For detailed analyses of MPO_2/5_-HLA-B7 binding properties, we generated structural models of the complexes and performed molecular dynamics (MD) simulations for a total time of 450 ns for each system. Figures 4A,B depict a representative MD conformation of MPO_2/5_-HLA-B7 complexes and Table 2 presents an alignment of both peptides according to structurally equivalent residue positions. Our results show that MPO_2_ features no structurally equivalent residue for W4 in MPO_5_ and that the TCR accessible residues are Q4, N6, V7 in MPO_2_, and W4, D5, E7, R8 in MPO_5_, respectively (Figures 4A,B; Table 2; violet residues). Hence, these residues are prone to affect TCR binding. Figures 4C,D highlight the steric and electrostatic properties of these solvent-exposed residues. All other MPO_2/5_ residues are oriented toward the HLA-B7 peptide binding cleft (Figures 4A,B; Table 2; orange residues). Of note, strong differences could be observed by analyzing the conformational stability of both peptides in the bound complex. MPO_5_ forms a stable complex structure throughout all MD simulations. MPO_2_, however, shows an overall stronger conformational flexibility, especially toward the N-terminus (Figures 4E,F), indicating a less stable binding to HLA-B7. This fits with our Molecular Mechanics/Generalized Born Surface Area (MM/GBSA)-based binding affinity estimations in which the affinity of MPO_5_ is stronger for HLA-B7 compared to MPO_2_ (Δ${\text{G}}_{\text{MMGBSA}}^{\text{MPO}5}$: −112.67 kcal mol^−1^ with σ = 20.55 and $\sigma_{\bar{\text{x}}}$ = 0.35, Δ${\text{G}}_{\text{MMGBSA}}^{\text{MPO}2}$: −88.02 kcal mol^−1^ with σ = 9.52 and $\sigma_{\bar{\text{x}}}$ = 0.16, σ: standard deviation, $\sigma_{\bar{\text{x}}}$: standard error of the mean). The superior binding of MPO_5_ can be explained by stronger enthalpic stabilization of the complex as it forms on average 2.25 hydrogen bonds more than MPO_2_ (Table 3; upper region). MPO_5_ is especially stabilized via hydrogen bonds of N1, R3, W4, and R8 compared to equivalent residues of MPO_2_ (lower region of Table 3; Supplementary Table 2). Particularly R3 of MPO_5_ is anchored strongly at the bottom of a deep pocket of the HLA-B7 peptide binding cleft via hydrogen bonds of its guanidino group with HLA-B7 residues S97 and D114 (see Figure 4B; green inlay). MPO_5_ shows pronounced flexibility only at position G6 (Figure 4E), which is strongly reduced by Ala- or Thr-exchange at this position (G6A or G6T; Supplementary Figure 4B, yellow inlay). This leads to stronger p-MHC binding affinities (Δ${\text{G}}_{\text{MMGBSA}}^{\text{G}6\text{A}}$: −112.94 kcal mol^−1^ with σ = 12.40 and $\sigma_{\bar{\text{x}}}$ = 0.20, Δ${\text{G}}_{\text{MMGBSA}}^{\text{G}6\text{T}}$: −118.28 kcal mol^−1^ with σ = 11.51 and $\sigma_{\bar{\text{x}}}$ = 0.19) as well as to higher IFN-γ release of TCR2.5D6-transduced T cells (27). In contrast, MPO_2_ binding is characterized by high flexibility distributed over several residues (Figure 4E). This multi-flexibility of the peptide correlates to the reduced HLA-stability seen in our UV-exchange and thermal shift assays and the weaker p-MHC binding affinity and constitutes one conceivable explanation for the reduced surface presentation, compensable by external peptide pulsing (Figure 1G) as well as the inferiority of TCRF5.4 in our *in vivo* experiments. However, structural features that might explain why MPO_2_ still binds well enough, are (i) its residue L5 (Figure 4A, green inlay) as it sterically fits into the central space of the HLA-B7 peptide binding cleft, (ii) N6, which forms stable hydrogen bonds with E152 (Table 3; Supplementary Table 2), and (iii) the central residues Q4 and L5 are located deeper in the HLA-B7 peptide binding cleft, leading to a stronger stabilization of these residues via hydrogen bonds (Table 3; Supplementary Table 2). Further MD simulations of HLA-B7 complexes comprising Ala/Thr variants of MPO_2/5_ supporting the effects described here are presented in Supplementary Figures 4A,B indicating that also TCR cross-reactivity may be implicated by peptide binding. Summarized, our p-MHC modeling strongly promotes our experimental results and revealed reasons for the weaker MPO_2_-HLA-B7 binding quality and in turn why MPO_5_ is superior in this regard.

**Table 2:** Alignment of the MPO_2_ octamer and the MPO_5_ nonamer peptides according to their HLA-B7-bound structures.

**MPO_**2**_**	**P1**	**P2**	**P3**		**P4**	**P5**	**P6**	**P7**	**P8**
	T	P	A		Q	L	N	V	L
**MPO**_**5**_	**P1**	**P2**	**P3**	**P4**	**P5**	**P6**	**P7**	**P8**	**P9**
	N	P	R	W	D	G	E	R	L

*Residues accommodated by the HLA-B7 peptide binding cleft are highlighted in orange. Residues with side chains oriented toward the solvent and thus accessible to T cell receptors (TCRs) are highlighted in purple. Residue positions are separately named for both peptides in consecutive order. As position P6 of MPO_5_ and P5 of MPO_2_ structurally bind into the same sub-pocket of the HLA-B7 peptide binding cleft they are considered equivalent*.

**Table 3:** Comparison of the average number of peptide-HLA-B7 binding cleft hydrogen bonds observed during the MD simulations for the bound MPO_2/5_ peptides.

**MPO**_****2****_-HLA-B*07:02		**MPO**_****5****_-HLA-B*07:02		**<hb>_MPO2_- < hb>_MPO5_**
**Entire peptide**	**<hb>**^a^	**Entire peptide**	**<hb>**	
SC^b^	1.13	SC	4.36	−3.23
BB^c^	7.51	BB	6.53	0.98
SC+BB	8.64	SC+BB	10.89	**−2.25**
**Individual peptide residues**		**Individual peptide residues**		
**P1**		**P1**		
T1_SC_	0.43	N1_SC_	1.14	−0.71
T1_BB_	1.26	N1_BB_	1.78	−0.52
T1_SC+BB_	1.69	N1_SC+BB_	2.92	**−1.23**
**P3**		**P3**		
A3_SC_	0.00	R3_SC_	2.01	−2.01
A3_BB_	0.66	R3_BB_	0.39	0.27
A3_SC+BB_	0.66	R3_SC+BB_	2.40	**−1.74**
**Missing residue**		**P4**		
/	0.00	W4_SC_	0.46	**−0.46**
**P4**		**P5**		
Q4_SC_	0.17	D5_SC_	0.23	−0.06
Q4_BB_	0.25	D5_BB_	0.04	0.21
Q4_SC+BB_	0.42	D5_SC+BB_	0.27	**0.15**
**P5**		**P6**		
L5_BB_	0.27	G6_BB_	0.11	**0.16**
**P6**		**P7**		
N6_SC_	0.53	E7_SC_	0.00	0.53
N6_BB_	0.22	E7_BB_	0.15	0.07
N6_SC+BB_	0.75	E7_SC+BB_	0.15	**0.60**
**P7**		**P8**		
V7_SC_	0.00	R8_SC_	0.52	−0.52
V7_BB_	0.61	R8_BB_	0.67	−0.06
V7_SC+BB_	0.61	R8_SC+BB_	1.19	**−0.58**
**P8**		**P9**		
L8_BB_	4.24	L9_BB_	3.39	**0.85**

*The upper part of the table compares the overall average number of hydrogen bonds for both peptides. The average number of hydrogen bonds are further subdivided in peptide side chain (SC)-mediated and peptide backbone (BB)-mediated hydrogen bonds. The lower part of the table compares the average number of hydrogen bonds for structurally equivalent residue positions in both peptides. Red: the MPO_2_ peptide forms less hydrogen bonds at a specific residue position compared to the MPO_5_ peptide, green: the MPO_2_ peptide forms more hydrogen bonds at a specific residue position compared to the MPO_5_ peptide. Only peptide residues that form SC- or BB-mediated hydrogen bonds with the HLA-B7 protein for at least one of the two peptides are listed and in these cases SC- or BB-mediated hydrogen bonds are always listed for both peptides*.

a Average number of hydrogen bonds per molecular dynamics time frame;

b SC, only side chain hydrogen bond interactions of the corresponding peptide or residue are considered;

c *BB, only backbone hydrogen bond interactions of the corresponding peptide or residue are considered*.

## Discussion

We here compared two MHC-mismatched allorestricted TCR with specificity for two different MPO-derived peptide ligands presented by HLA-B7 and identified by MS immunopeptidomic analyses of tumor samples derived from patients with MPN. Such TCR may be used in the context of allogeneic stem cell transplantation in a haplo- or sHLAm setting as well as alternatively within a conditioning regimen using TCR-transgenic T cells. A novel allorestricted TCR (TCRF5.4) demonstrating high specificity for a new HLA-B7-restricted MPO_2_-derived peptide was compared by in-depth functional characterization with the previously identified TCR2.5D6 (27) in order to define characteristics essential for effective tumor rejection. In fact, both TCRs differed strongly in their tumor rejection capacity in our long-term *in vivo* experiments using transduced T cells with comparable TCR expression demonstrating TCR2.5D6 to be clearly superior to TCRF5.4. The group receiving TCRF5.4-transgenic T cells was not able to completely reject the tumor and experienced an early relapse. In contrast, tumor eradication was more effective in the group of TCR2.5D6, although also these animals relapsed. However, tumor escape mechanisms were different in both groups. Whereas, tumors in mice treated with TCR2.5D6-transduced T cells lacked HLA-B7 and lost all TCR-transgenic T cells, TCRF5.4-transgenic T cells could be detected in all examined tissues except in tumors with preserved HLA-B7 expression. Loss of HLA-B7 indicates the immunogenic pressure induced by TCR2.5D6-transduced T cells. In contrast, although TCRF5.4-transgenic T cells have well engrafted, our experiments indicate that tumor escape relies more on peptide and/or TCR-intrinsic qualities resulting in T-cell exclusion from the tumor.

As *in vivo* experiments are highly laborious, it is desirable to define *in vitro* assays most effective in predicting *in vivo* activity. Numerous *in vitro* assays have been proposed to reflect strength of T-cell response correlating with tumor rejection capacity *in vivo* (8–10, 12, 17, 18, 63) although these analyses have been mainly tested in the autologous setting. In fact, many of these TCR-dependent functional assays showed comparable results for both TCR in our experiments and therefore failed to predict *in vivo* outcome in the allorestricted setting.

Focusing on differences between our allorestricted TCRs we observed in almost all TCR transductions reduced quantitative and qualitative surface expression for TCRF5.4. Although the quality of TCR expression is critical for T cell performances and endogenous TCR chains can influence the function and presentation of transgenic TCR (16), the TCRF5.4 represents a generally well-expressed TCR. Furthermore, for the detailed comparisons of both TCRs we used freshly isolated T cell subpopulations from different healthy donors for several reasons. (i) First, this minimize donor-dependent effects of TCR-transgenic T cells that would rule out general statements for the observed functional qualities. (ii) Second, the use of freshly isolated donor T cells ensures optimal T-cell fitness and reduces the impact of T-cell exhaustion because of freeze and thaw cycles, strenuous selection processes, and/or too long culture periods. (iii) Third, large numbers of TCR-transductions using different donor T cells give the opportunity to estimate possible donor-related heterogeneity in results or, as observed in our case, a reliable robustness and consistency of T cells transduced by TCRF5.4 and TCR2.5D6. Furthermore, TCR-transgenic T cells and HLA-transgenic tumor cell lines were used without additional adjustment of TCR- or antigen quality after transduction. Hence, our results include individual characteristics of both TCRs that would also be present in clinical settings in the T-cell products for adoptive transfers. However, for our significant long-term *in vivo* experiments we selected T-cell products for both TCR with comparable quantitative and qualitative TCR expression and still observed inferior tumor rejection capacity of TCRF5.4-transduced T cells. These results indicate that differences in TCR expression may not represent a decisive criterion for TCR selection in our case. Especially, we could not confirm any correlation of *in vivo* anti-tumor reactivity to dissociation half-lives of TCRs from their p-MHC complexes (12, 64) as well as functional avidity of TCRs toward the cognate MHC-peptide complexes (9). This could be explained by specific features of MHC-mismatched TCRs as they have alternative binding properties to the mismatched MHC (65) compensating for lower affinity of the peptide toward MHC as well as the TCR toward peptide.

Differences in quantitative and qualitative cytokine secretion, however, revealed to be the most significant indicator for reduced *in vivo* performance seen in almost all *in vitro* analyses with differing but also equal TCR expression potentially explaining inferiority in tumor rejection (66, 67). In contrast, TCRF5.4-transduced T cells were competitive to those transduced with TCR2.5D6 with respect to *in vitro* cytotoxicity being in line with previous observations that this functional quality may not represent the most sensitive test for T-cell responses even with differing TCR expression rates (68) indicating once more that TCR expression is not the most indicative factor for TCR functionality.

In contrast, our data support that peptide presentation on the surface of tumor cells may represent a key factor for effective tumor rejection. In fact, although the MPO antigen expression in this work was every time identically for both TCR conditions as the targeted tumor cell lines were the same, the MPO-derived target peptide presentation for MPO_2_ and MPO_5_ was obviously different. Our results indicate a reduced peptide binding of MPO_2_ to HLA-B7 when comparing both peptides by either UV exchange assays, thermal shift assays or predicted HLA-affinity suggesting that reduced peptide binding toward HLA-B7 plays a major role in inferior tumor rejection. Although the absolute quantification of peptides on the surface of target cells by MS harbors major limitations, our MS data may support an inferior MHC presentation of MPO_2_ compared to MPO_5_ due to its highly reduced detection. Our data thereby confirm data from Engels et al. highlighting the role of peptide binding to tumor rejection (18), here in an MHC-mismatched setting.

To further deepen our understanding of p-MHC binding, we applied structure-based modeling and atomistic MD simulations and realized the potential of molecular modeling and simulation to rationalize the results of our peptide stability and amino acid substitution assays on a molecular level. We thereby not only confirmed UV exchange assays as well as sequence-based affinity prediction but also discovered MPO_5_ to be more rigid within the peptide binding cleft due to better stabilization of amino acid residues by hydrogen bonds at individual positions in comparison to MPO_2_. This finding, in fact, may be decisive for a high specificity of a selected TCR as more flexible amino acid residue positions of the peptide may impede stable binding of the TCR to peptide MHC. Indeed, we can explain by Cα atoms fluctuations especially for the MPO_5_ peptide that the binding quality of respective amino acid residues in their HLA-binding pocket favors a more stable peptide binding. The high flexibility in position 6 is markedly reduced after exchange by alanine or threonine indicating stabilization of the peptide toward HLA-B7. Importantly, reactivity of the TCR does not depend on the glycine within the wildtype peptide and is even enhanced after stabilization of the peptide by alanine or threonine. In contrast, the enhanced fluctuation observed for several amino acid residues of the MPO_2_ peptide correlates with overall less formed hydrogen bonds between the p-MHC complex compared to MPO_5_ and more flexibility of p-MHC recognition within the alanine and threonine scan. Taken together, our data extend previous publications (69) and indicate, that for cross-reactivity testing at least two different amino acids for amino acid substitution assays are necessary to assess possible TCR-binding motifs.

Moreover, there seems to be a correlation between the described flexibility of p-MHC binding as analyzed by structure-based modeling and the cross-reactivity of TCRF5.4 as identified by its observed flexibility to amino acids exchanges of the peptide, the high number of peptides predicted to be recognized by TCRF5.4 by ScanProsite as well as its MHC-cross-reactivity toward HLA-B51 which is in contrast to the observations made for TCR2.5D6. In this regard the rigidness evaluation of peptide-HLA binding might have the potential to serve as an appropriate tool to predict cross-reactivity. Although structure-based modeling of p-MHC complexes can provide valuable insights into the molecular basis of TCR-p-MHC recognition, this strongly depends on the availability of experimental structural data of p-MHC either of the HLA to be investigated or of close homologs. As the latter is often the case for many MHC proteins, homology modeling approaches can nowadays be applied to construct MHC protein models for most HLA-alleles. Bound peptides can be modeled via side chain placement/mutation algorithms using MHC-bound peptide backbone coordinates from X-ray structures as template. As peptide binding is determined by the structural and chemical characteristics of the binding cleft of the specific MHC allele, binding features have to be analyzed on an allele-specific basis. For this, standard analysis approaches, which are readily available for most MD-simulation packages, can be applied, such as hydrogen bond analysis (for, e.g., hydrophilic binding pockets) or residue contact maps (for, e.g., hydrophobic binding pockets) or combinations thereof.

**Figure 5:**
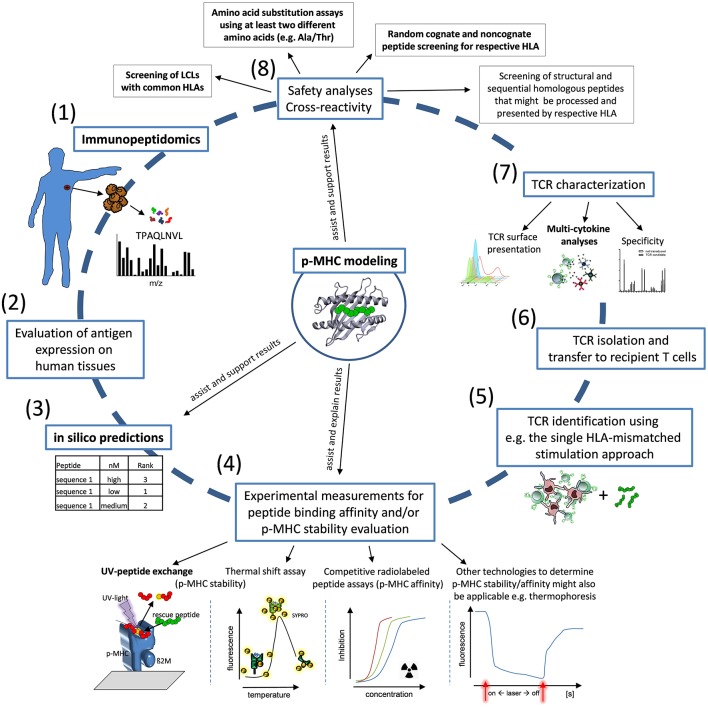
Workflow for identification of allo-MHC-restricted tumor-reactive TCRs. Proposed workflow for TCR identification: (1) Immunopeptidomics of patient samples and tumor material; (2) Evaluation of antigen expression on human tissues; (3,4) *in silico* prediction of candidate peptides for first ranking and experimental validation by HLA-affinity and/or HLA-stability assays in combination with p-MHC modeling; (5,6) TCR identification and TCR isolation; (7) TCR characterization with special interest to TCR surface presentation, cytokine secretion and specificity; (8) Characterization of the safety profile of TCR-transgenic T cells using LCL cell lines, amino acid substitution experiments and screening of homologous peptides in combination with structure-based modeling approaches. Critical assays for the identification of suitable TCR and peptides used in our analyses are highlighted in bold characters.

Although the number of TCRs studied here is limited and more TCR candidates need to be tested to further prove our conclusions, our integrated view in TCR functionality and p-MHC binding suggests the quality and quantity of cytokine release and the characteristics of p-MHC binding as most relevant factors to be analyzed for a fast and efficient TCR identification procedure in the allogeneic MHC-mismatched repertoire. We conclude that an interdisciplinary approach between *in silico*-, T-cell-, and structural analyses is most promising to address the challenges of identification of TCR with high efficacy and safety profiles. We propose a curtailed procedure to identify allorestricted TCRs presumably also applicable to be used for other settings as for autologous TCR specific for other tumor-associated antigens and neoantigens (Figure 5). The final aim of this workflow is to identify efficiently TCR candidates for clinical translation to enable personalized immunotherapies with high tumor reactivity and a favorable safety profile.

## Ethics Statement

This study was carried out in accordance with the recommendations of conventional institute guidelines. The protocol was approved by the local authorities.

## Author Contributions

SA and AK: study design and conception together with RK, EB, and SM. SM and SA: *in vivo* characterization studies and animal experiments. RK, EB, and SA: *in vitro* characterization studies. MP and MM: mass spectrometry studies. MG and IA: protein modeling and *in silico* MPO-peptide characterization. ME and DB: TCR k_off_-rate measurements, provision of HLA-multimers. SM, SA, HB, NY, and EB: experimental support in subcutaneous and i.v. injection of mice, maintenance of mice. JP and SS: experimental support UV-exchange assay, provision of UV-sensitive HLA-monomers. SA, MP, MG, IA, and AK: manuscript writing. SA, EB, RK, SM, JA, ME, MP, JP, MG, HB, NY, SS, DB, MM, IA, and AK: manuscript preparation and review process. AK: study supervision.

### Conflict of Interest Statement

The authors declare that the research was conducted in the absence of any commercial or financial relationships that could be construed as a potential conflict of interest.

## Abbreviations

B2M: beta-2-microglobulin
EC_50_: half maximal effective concentration
IVT: *in vitro* transcribed
k_off_-rate: dissociation half-live of TCR from p-MHC complex
MD simulation: molecular dynamics simulation
MFI: mean fluorescence intensity
RMSD: root mean square deviation
RMSF: root mean square fluctuations
sHLAm: single HLA-mismatched approach
T_CM_: T cells enriched for the central memory phenotype
TCRm: mouse TCR-β chain.
