# Supplementary material for: Key Features Relevant to Select Antigens and TCR From the MHC-Mismatched Repertoire to Treat Cancer
Source: Front Immunol. 2019 Jun 28;10:1485. doi: 10.3389/fimmu.2019.01485 (PMC6611213; doi:10.3389/fimmu.2019.01485)
Supplement: Supplementary file 1 [file Data_Sheet_1.docx]

Supplementary Material

Key features relevant to select antigens and TCR from the MHC-mismatched repertoire to treat cancer

**Audehm S., Glaser M., Pecoraro M., Bräunlein E., Mall S., Klar R., Effenberger M., Albers J., Bianchi H.O., Peper J., Yusufi N., Busch D.H., Stevanović S., Mann M., Antes I., Krackhardt A.M.**

**Correspondence:** Krackhardt A.M: [Angela.Krackhardt@tum.de](mailto:Angela.Krackhardt@tum.de)

# Supplementary Figures and Tables

## Supplementary Figures

**C**

**B**

**A**


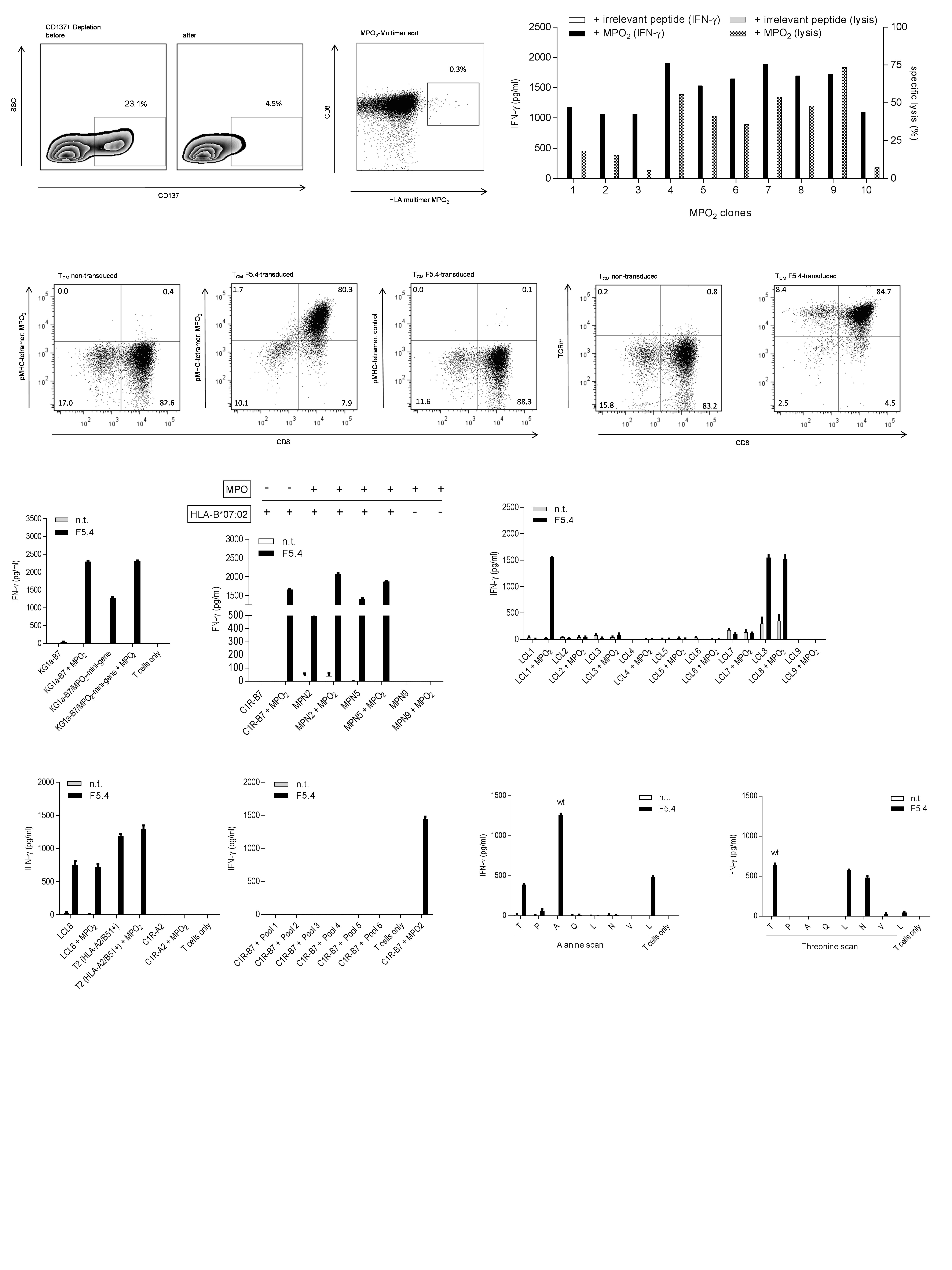


**K**

**J**

**I**

**H**

**E**

**F**

**G**

**D**

**Supplementary Figure 1.** **Identification and characterization of TCRF5.4 targeting MPO_2_-HLA-B7**

**(A)** CD137 depletion of alloreactive T cells after co-cultivation with lethally irradiated HLA-B7-transgenic C1R cells. CD137 antibody staining of pre-gated CD3^+^ cells is shown before and after depletion. **(B)** MPO_2_-specific HLA-multimer population selected for sorting is shown after stimulation of naïve T cells with MPO_2_-pulsed dendritic cells form the same donor electroporated with in vitro transcribed RNA (IVT-RNA) coding for HLA-B7. **(C)** IFN-γ release (left Y-axis) and cytotoxicity (right Y-axis) of MPO_2_-specific T-cell clones after co-cultivation with K562-HLA-B7eGFP cells at an E/T ratio of 20:1. IFN-γ release was analyzed after 20h of co-cultivation by ELISA and cytotoxicity after 45h by calculating eGFP-expression of remaining CD3^-^ target cells. **(D)** TCRF5.4 expression in recipient CD8^+^ T_CM_ after retroviral transfer. CD8^+^ T_CM_ transgenic for TCRF5.4 and non-transduced T cells were either stained with MPO_2_-specific HLA-multimer or control multimer (left set of panels) or anti-TCRm antibody (right set of panels) (n=3). **(E)** MPO_2_-speciﬁc IFN-γ release by TCRF5.4-transduced CD8^+^ T_CM_ in response to the MPO-negative KG1a-B7 either pulsed with MPO_2_ (+MPO_2_), transduced with the MPO_2_-mini-gene (/MPO_2_-mini-gene) or transduced with the MPO_2_-mini-gene with additional MPO_2_ pulsing (n=2). **(F)** IFN-γ release by TCRF5.4-transduced PBMC in response to malignant cells derived from patients with MPO-expressing myeloproliferative neoplasia (MPN) with or without HLA-B7 expression. C1R-B7 cells served as negative control (n=3). HLA characteristics of MPN patients are included in Table S2. **(G)** Investigation of allo-HLA reactivity of TCRF5.4-transduced PBMCs co-cultured with LCLs expressing 24 common HLA alleles within the Caucasian population with and without MPO_2_-peptide pulsing. HLA-B7-expressing LCL1 served as a positive control (n=4). HLA-characteristics are included in Supplementary Table 1. **(H)** IFN-γ release by TCRF5.4-transduced PBMC is shown for HLA-A2/B51^+^ T2 or HLA-A2-transgenic C1R (C1R-A2) either pulsed with MPO_2_ or without peptide pulsing. HLA-B51^+^ LCL8 were used as positive control (n=2). **(I)** IFN-γ release by TCRF5.4-transduced PBMC against a set of 58 HLA-B7 peptide ligands tested in pool stimulation loaded on C1R-B7 cells. C1R-B7 pulsed with MPO_2_ served as a positive control (n=2). **(J, K)** Peptide-specificity of TCRF5.4 tested by alanine- (n=4) (J) or threonine (n=6) (K) exchanges of any single amino acid of MPO_2_. Respective peptides were loaded on HLA-B7^+^ LCL1, wt = wildtype amino residue. (D-K) Non-transduced PBMC were used as negative controls and (E-K) standard deviation of the mean of triplicates are shown. An E/T ratio of 1:1 was used and IFN-γ secretion of the supernatants was measured by IFN-γ ELISA.

**B**

**A**


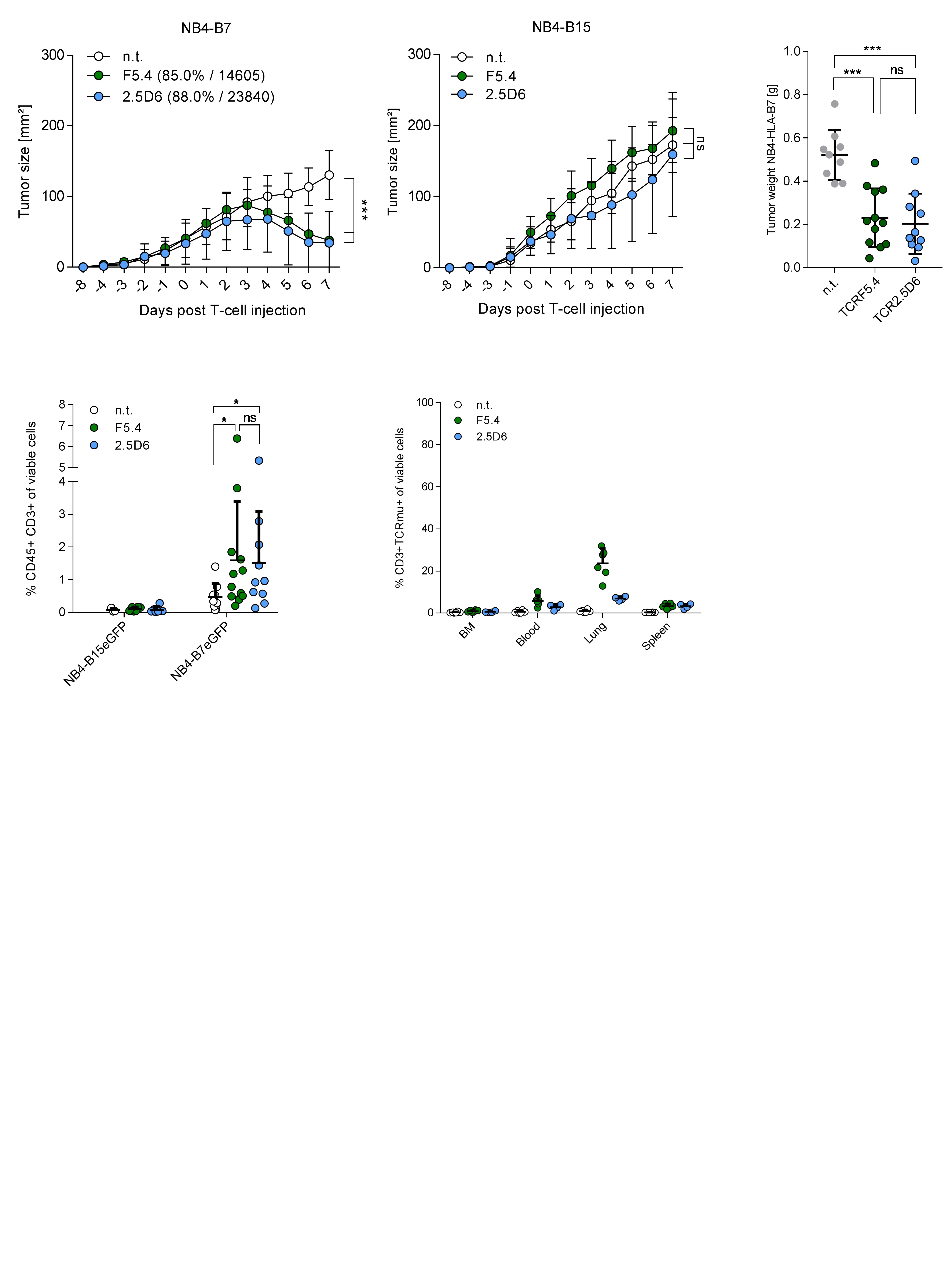


**D**

**C**

**Supplementary Figure 2. Short-term in vivo experiments revealed no major differences in tumor killing capacity between TCRF5.4 and TCR2.5D6**

**(A)** Tumor size in mm^2^ at defined days is shown for NB4-B7eGFP (left) or NB4-B15eGFP (right) derived tumors for the three different groups receiving either TCRF5.4-, TCR2.5D6- or non-transduced (n.t.) CD8^+^ T_CM_. Each group of NB4-B7eGFP tumors comprises 13 mice. The group of NB4-B15eGFP tumors comprises 6-7 mice, ^ns^p≥0.05, ***p≤0.0006. **(B)** Tumor weight of ex vivo analyzed NB4-B7eGFP tumors for n.t. (n=10), TCRF5.4 (n=12), TCR2.5D6 (n=10), ^ns^p≥0.05, ***p≤0.0003. **(C)** Percentage of CD45^+^/CD3^+^ T cells analyzed by flow cytometry of ex vivo analyzed NB4-B7eGFP- or NB-B15eGFP tumors for n.t. (n=9), TCRF5.4-transduced (n=12), TCR2.5D6-transduced (n=10), ^ns^p≥0.05, *p≤0.035. **(D)** Percentage of CD3^+^/TCRm^+^ viable cells in bone marrow (BM), blood, lung and spleen analyzed ex vivo by flow cytometry at day 7 after T-cell injection. N.t. (n=6), TCRF5.4-transduced (n=6) or TCR2.5D6-transduced T cells (n=4). (A-D) Pooled data of two different (n=2) experiments and the mean together with standard deviation is shown in each experiment.

**C**

**B**

**A**


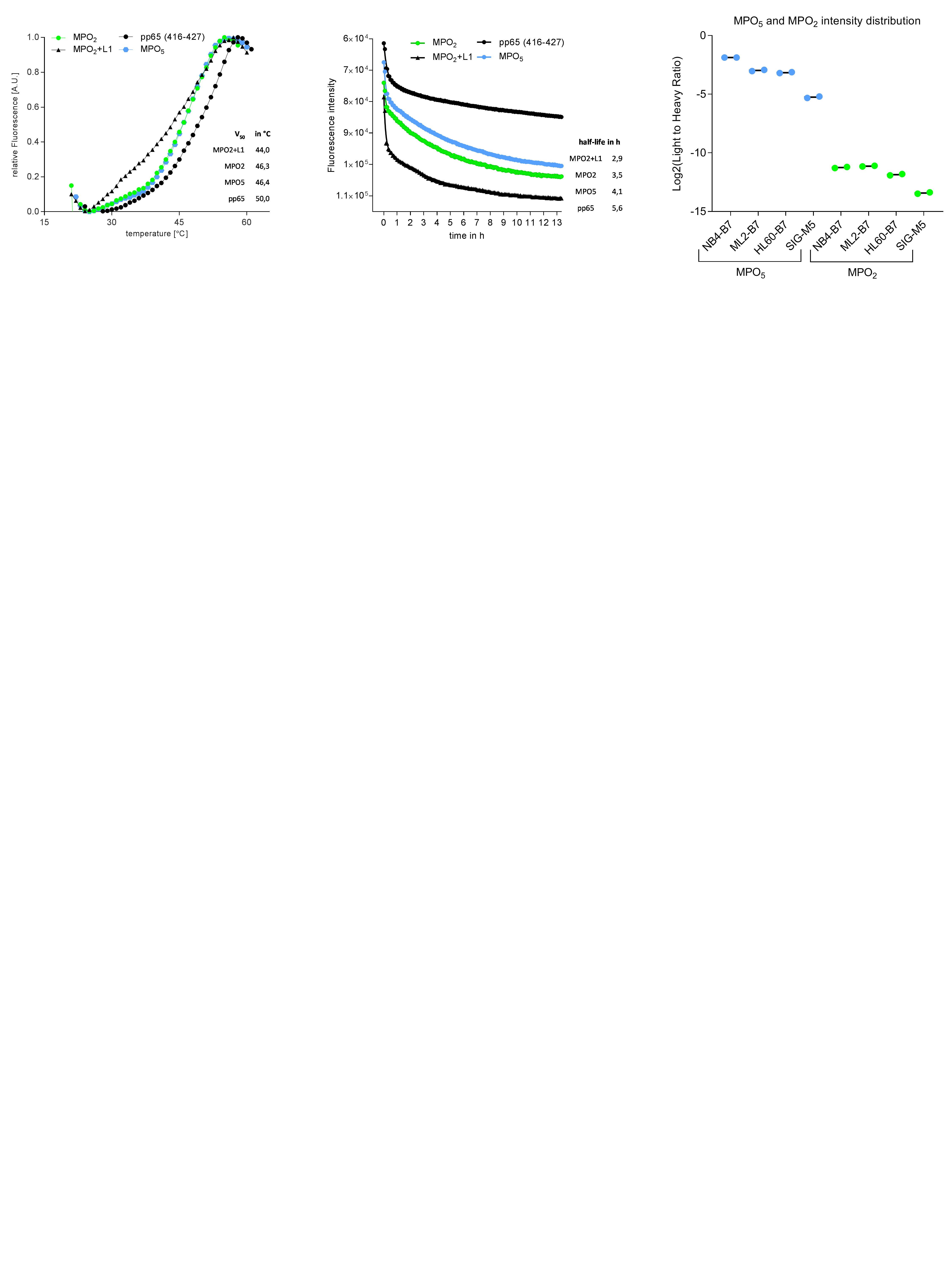


**Supplementary Figure 3. Compared to the MPO_5_-HLA-B7 the MPO_2_-HLA-B7 complex cannot compete with respect to thermal stability and detectability on the surface of different AML cell lines.**

**(A)** Thermal shift assay of MPO-peptides in comparison to pp65 and MPO_2_+L1. Boltzmann fitting was used to determine V_50_ values (half-maximal denaturation) for each p-MHC complex after gradual heating from 21°C to 95°C. The mean of quadruplicates is shown (n=2). **(B)** Measurement of kinetic stability of indicated p-MHC complexes in comparison to pp65 and MPO_2_+L1 at constant temperature of 37°C. For the determination of half-maximal decay of p-MHC complexes, the slowest constant rates were used after fitting the fluorescence values with an exponential two-phase decay equation. The mean of quadruplicates is shown (n=2). (A,B) The fluorescence was measured by monitoring the intensity of SYPRO orange protein dye binding to hydrophobic core regions of unfolded p-MHC-complexes. **(C)** Occurrence of the MPO_2_ and MPO_5_ peptides on the surface of indicated AML cell lines with endogenous HLA-B7 expression using the heavy labelled Leu7 counterpart for detection. Logarithmic intensities of MPO peptide surface presentation for MPO peptides are shown in duplicates. MPO_5_ peptides are depicted in blue, MPO_2_ peptides are depicted in green (n=2).

**B**

**A**

**Supplementary Figure 4. Structure-based modeling of alanine and threonine (Ala/Thr) variants of MPO_2/5_ bound to HLA‑B7 gave insights into MPO-peptide binding quality.**

**(A, B)** Representative conformation of MPO_2/5_ in the bound state. The HLA‑B7 protein is shown as cartoon or smooth molecular surface representation (grey), the bound peptide backbones are depicted in trace/cartoon representation (grey/orange/violet). The side chains of the HLA-B7-accommodated residues (orange carbon atoms) and their corresponding Ala/Thr mutations (violet carbon atoms) are shown as sticks and in atom type coloring (hydrogen atoms: white, nitrogen atoms: blue, oxygen atoms: red, carbon atoms: orange or violet). (A) Binding features of the MPO_2_ residues P2 (blue inlay), A3 (red inlay), L5 (yellow inlay), and L8 (green inlay) bound to the HLA-B7 protein and the structural impacts of Ala/Thr mutations of these residue positions are emphasized. Data for position 1 and the TCR-accessible residue positions 4, 6 and 7 are not shown. Blue inlay: focus on the HLA-B7 pocket accommodating P2 (space filling model). P2 is sterically optimally filling the space enclosed by the corresponding HLA‑B7 pocket and thus serves as anchor residue for the peptide (see also Immune Epitope Database and Analysis Resource (IEDB) Peptide MHC Binding Motif ([Vita et al., 2015](#_ENREF_67)))_._ Red inlay: direct comparison of the binding mode of the wildtype MPO_2_ residue A3 and of the corresponding Thr mutant. The HLA-B7 residues forming the deep binding pocket accepting the side chain of the residue at position 3 are highlighted in blue stick representation and transparent smooth molecular surface. Additionally, the residue D114 is shown (blue sticks), which is located at the bottom of the binding pocket. The residue L5 is depicted (pastel-coloring) for direct comparison with the binding mode of G6 in MPO_5_. The side chain of threonine is too short to stably interact with the HLA-B7 residue D114 (as for example R3 in MPO_5_) and its polar side chain is not stably bound by the upper, nonpolar part of the pocket. Therefore, it is preferentially located outside of the pocket, leading to a lifted position of the entire residue towards the solvent in the MD simulation. Yellow inlay: impact of Ala/Thr mutations of the residue L5. A3 (pastel-coloring) is shown for direct comparison with the binding mode of R3 in MPO_5_. L5 is accommodated by the central volume of the HLA-B7 peptide binding cleft. As this part of the HLA-B7 binding cleft is amphipathic, it can also accommodate the polar residue T5. Its polar side chain is partially stabilized by hydrogen bonds to residues in the MHC peptide binding cleft. The small methyl side chain of A5 is less space-consuming and not optimally filling the volume of the central region of the HLA-B7 peptide binding cleft compared to the more voluminous side chain of L5 in the wildtype. The L5A variant of MPO_2_ misses both, the beneficial anchoring residue R3 and a space-consuming residue at position 5, capable of optimally filling the central volume of the HLA-B7 peptide binding cleft and therefore compensating for missing R3. Green inlay: focus on the HLA-B7 pocket accommodating L8 and the binding mode of the corresponding L8A and L8T MPO_2_ variants. L8 serves as an anchor residue (IEDB). The HLA-B7 pocket accommodating residue position 8 exhibits a nonpolar surface, especially towards the bottom of the pocket. With the side chain of leucine occupying this pocket, nonpolar packing is optimized. The side chains of Ala/Thr are either too small or too polar for optimal nonpolar packing. (B) Binding features of the MPO_5_ residues P2 (blue inlay), R3 (red inlay), G6 (yellow inlay), and L9 (green inlay) and the structural impact of Ala/Thr mutations of these residue positions are emphasized. Data for position 1 and the TCR-accessible residue positions 4, 5, 7 and 8 are not shown. Blue inlay: focus on the HLA-B7 pocket accommodating P2 (space filling model) of MPO_5_. As for MPO_2_, P2 serves as an anchor residue (vide supra). Red inlay: binding mode of R3 of MPO_5_ and of the corresponding R3A and R3T MPO_5_ variants. The HLA-B7 residues D114 and S97 stabilize R3 in the HLA-B7 binding cleft via hydrogen bonding and are highlighted in blue stick representation. Hydrogen bonds are emphasized for the representative conformation in magenta. The residue G6 (stick representation, pastel-coloring) is shown for direct comparison with the binding mode of L5 in MPO_2_. Arginine is the only preferred residue at position 3 (IEDB). The methyl group of A3 cannot form hydrogen bonds and T3 is too short to reach the residues D114 and S97 at the bottom of the HLA-B7 peptide binding cleft. Yellow inlay: impact of G6A and G6T mutations on the HLA-B7 binding mode of wildtype MPO_5_ (WT). The representatives of the four biggest conformational clusters of peptide ligand backbone conformations sampled via MD are depicted in cartoon representation. The representative of the biggest cluster is colored in orange (WT: 62.40 %, G6A: 69.60 %, G6T: 66.60 %), the representative of the second biggest cluster is colored in violet (WT: 33.30 %, G6A: 18.60 %, G6T: 23.50 %), the representative of the third biggest cluster is colored in blue (WT: 4.20 %, G6A: 7.70 %, G6T: 8.80 %), and the representative of the fourth biggest cluster in green (WT: 0.10 %, G6A: 3.30 %, G6T: 0.60 %). Additionally, the residues R3, D5, G6/A6/T6 of the representative of the biggest peptide ligand backbone conformational cluster are shown as sticks (orange). Intra-peptide hydrogen bonds are indicated in magenta. G6 in the HLA-B7-bound wildtype MPO_5_ shows a high flexibility in the MD simulations, leading to overall peptide conformations with a kinked side chain position of D5. Due to stabilization of position 6 through additional interactions of the Ala and Thr side chains with the HLA-B7 peptide binding cleft in the G6A and G6T variant-HLA-B7 complexes, position 6 is less flexible in these variants. This leads to a conformation of the HLA-B7-bound peptide in which D5 protrudes into the solvent. Therefore, D5 has a stronger TCR-exposed conformation in the G6A and G6T variant-HLA-B7 complexes compared to the HLA-B7-bound wildtype MPO_5_. Green inlay: focus on the HLA-B7 pocket accommodating L9 of MPO_5_ and the binding mode of the corresponding L9A and L9T MPO_5_ variants. L9 is accommodated by the same pocket as L8 in MPO_2_ and serves as anchor residue (vide supra).

## Supplementary Tables

**Supplementary Table 1: HLA-alleles of LCL cell lines, AML cell lines and MPN samples**

| Cell line | HLA-A* | HLA-B* |
| --- | --- | --- |
| ML2 | 02:01/- | 44:02/51:01 |
| SiG-M5 | 24:02/01:01 | 51:01/07:02 |
| T2 | 02:01/- | 51:01/- |
| NB4 | 11:01/- | 35:01/40:01 |
| HL60 | 01:01/- | 57:01/- |
| C1R | lacking surface HLA-A* and B* antigens | |
| LCL1 | 02:01/ -- | 07:02/15:01 |
| LCL2 | 03:01/ -- | 27:05/ -- |
| LCL3 | 29:02/ -- | 40:02/ -- |
| LCL4 | 02:17/ -- | 15:01/ -- |
| LCL5 | 02:01/03:01 | 35:02/38:01 |
| LCL6 | 68:02/30:01 | 42:01/ -- |
| LCL7 | 01:01/02:08 | 08:01/50:01 |
| LCL8 | 02:04/ -- | 51:01/ -- |
| LCL9 | 01:01/ -- | 41:01/ -- |
| LCL10 | 01:01/ 02:01 | 07:02/40:01 |
| MPN 2 | 03:01/25:01 | 07:02/15:01 |
| MPN 5 | 01:01/33:01 | 07:02/14:02 |
| MPN 9 | not defined; | negative for B*07:02 |

**Supplementary Table 2. Comparison of the average number of peptide-HLA-B7 binding cleft hydrogen bonds observed during the molecular dynamics (MD) simulations for residues at structurally equivalent positions in the bound MPO_2_ and MPO_5_ peptides (see Table 2 and 3).**

The table is an extended version of Table 3, additionally listing the specific HLA‑B7 residues that are interacting with MPO_2/5_ residues via hydrogen bonds. Only peptide residue positions that show hydrogen bond interactions with the HLA‑B7 protein for at least one of the two peptides are listed. Specific hydrogen bond interactions are always listed for both peptides, even if they show no occurrence for one of them.

| **MPO_5_-HLA-B*07:02** | | **MPO_2_-HLA-B*07:02** | | **<hb>_MPO2_-<hb>_MPO5_** |
| --- | --- | --- | --- | --- |
| **Residue Pair** | **<hb>^a^** | **Residue Pair** | **<hb>** |  |
| **Position 1** | | **Position 1** | |  |
| N1_SC_^b^-Y171 | 0.52 | T1_SC_-Y171 | 0.00 | -0.52 |
| N1_SC_-E163 | 0.45 | T1_SC_-E163 | 0.34 | -0.11 |
| N1_SC_-N63 | 0.11 | T1_SC_-N63 | 0.05 | -0.06 |
| N1_SC_-Y59 | 0.06 | T1_SC_-Y59 | 0.04 | -0.02 |
| *Sum_SC_* | *1.14* | *Sum_SC_* | *0.43* | *-0.71* |
| N1_BB_^c^-Y171 | 0.03 | T1_BB_-Y171 | 0.01 | -0.02 |
| N1_BB_-E163 | 0.00 | T1_BB_-E163 | 0.02 | 0.02 |
| N1_BB_-Y159 | 0.93 | T1_BB_-Y159 | 0.58 | -0.35 |
| N1_BB_-Y67 | 0.00 | T1_BB_-Y67 | 0.02 | 0.02 |
| N1_BB_-Y59 | 0.28 | T1_BB_-Y59 | 0.10 | -0.18 |
| N1_BB_-E45 | 0.29 | T1_BB_-E45 | 0.20 | -0.09 |
| N1_BB_-Y9 | 0.00 | T1_BB_-Y9 | 0.17 | 0.17 |
| N1_BB_-Y7 | 0.25 | T1_BB_-Y7 | 0.16 | -0.09 |
| *Sum_BB_* | *1.78* | *Sum_BB_* | *1.26* | *-0.52* |
| **Sum_SC+BB_** | **2.92** | **Sum_SC+BB_** | **1.69** | **-1.23** |
| **Position 3** | | **Position 3** | |  |
| R3sc-Y116 | 0.02 | A3sc-Y116 | 0.00 | -0.02 |
| R3sc-D114 | 1.68 | A3sc-D114 | 0.00 | -1.68 |
| R3sc-S97 | 0.31 | A3sc-S97 | 0.00 | -0.31 |
| *Sum_SC_* | *2.01* | *Sum_SC_* | *0.00* | *-2.01* |
| R3_BB_-Y99 | 0.39 | A3_BB_-Y99 | 0.33 | -0.06 |
| R3_BB_-Q70 | 0.00 | A3_BB_-Q70 | 0.10 | 0.10 |
| R3_BB_-Y9 | 0.00 | A3_BB_-Y9 | 0.23 | 0.23 |
| *Sum_BB_* | *0.39* | *Sum_BB_* | *0.66* | *0.27* |
| **Sum_SC+BB_** | **2.40** | **Sum_SC+BB_** | **0.66** | **-1.74** |
| **Position 4** | | **Missing residue** | |  |
| W4_SC_-E163 | 0.44 | / | 0.00 | -0.44 |
| W4_SC_-Q155 | 0.02 | / | 0.00 | -0.02 |
| **Sum_SC_** | **0.46** | **Sum_SC_** | **0.00** | **-0.46** |
| **Position 5** | | **Position 4** | |  |
| D5_SC_-R156 | 0.11 | Q4_SC_-R156 | 0.00 | -0.11 |
| D5_SC_-Q155 | 0.06 | Q4_SC_-Q155 | 0.04 | -0.02 |
| D5_SC_-T73 | 0.06 | Q4_SC_-T73 | 0.00 | -0.06 |
| D5_SC_-Q70 | 0.00 | Q4_SC_-Q70 | 0.13 | 0.13 |
| *Sum_SC_* | *0.23* | *Sum_SC_* | *0.17* | *-0.06* |
| D5_BB_-Q155 | 0.04 | Q4_BB_-Q155 | 0.25 | 0.21 |
| *Sum_BB_* | *0.04* | *Sum_BB_* | *0.25* | *0.21* |
| **Sum_SC+BB_** | **0.27** | **Sum_SC+BB_** | **0.42** | **0.15** |
| **Position 6** | | **Position 5** | |  |
| G6_BB_-T73 | 0.03 | L5_BB_-T73 | 0.00 | -0.03 |
| G6_BB_-Q70 | 0.08 | L5_BB_-Q70 | 0.27 | 0.19 |
| **Sum_BB_** | **0.11** | **Sum_BB_** | **0.27** | **0.16** |
| **Position 7** | | **Position 6** | |  |
| E7_SC_-E152 | 0.00 | N6_SC_-E152 | 0.53 | 0.53 |
| *Sum_SC_* | *0.00* | *Sum_SC_* | *0.53* | *0.53* |
| E7_BB_-E152 | 0.15 | N6_BB_-E152 | 0.22 | 0.07 |
| *Sum_BB_* | *0.15* | *Sum_BB_* | *0.22* | *0.07* |
| **Sum_SC+BB_** | **0.15** | **Sum_SC+BB_** | **0.75** | **0.60** |
| **Position 8** | | **Position 7** | |  |
| R8_SC_-E189 | 0.24 | V7_SC_-E189 | 0.00 | -0.24 |
| R8_SC_-E76 | 0.26 | V7_SC_-E76 | 0.00 | -0.26 |
| R8_SC_-T73 | 0.02 | V7_SC_-T73 | 0.00 | -0.02 |
| *Sum_SC_* | *0.52* | *Sum_SC_* | *0.00* | *-0.52* |
| R8_BB_-W147 | 0.64 | V7_BB_-W147 | 0.56 | -0.08 |
| R8_BB_-K146 | 0.03 | V7_BB_-K146 | 0.05 | 0.02 |
| *Sum_BB_* | *0.67* | *Sum_BB_* | *0.61* | *-0.06* |
| **Sum_SC+BB_** | **1.19** | **Sum_SC+BB_** | **0.61** | **-0.58** |
| **Position 9** | | **Position 8** | |  |
| L9_BB_-W147 | 0.00 | L8_BB_-W147 | 0.56 | 0.56 |
| L9_BB_-W146 | 0.73 | L8_BB_-146 | 0.82 | 0.09 |
| L9_BB_-T143 | 0.98 | L8_BB_-143 | 0.98 | 0.00 |
| L9_BB_-Y84 | 0.82 | L8_BB_-Y84 | 0.92 | 0.10 |
| L9_BB_-N80 | 0.70 | L8_BB_-N80 | 0.85 | 0.15 |
| L9_BB_-S77 | 0.16 | L8_BB_-S77 | 0.11 | -0.05 |
| **Sum_BB_** | **3.39** | **Sum_BB_** | **4.24** | **0.85** |

^a^Average number of hydrogen bonds per molecular dynamics time frame.

^b^SC: only side chain hydrogen bond interactions of the corresponding residue are considered.

^c^BB: only backbone hydrogen bond interactions of the corresponding residue are considered.

## Supplementary Methods

**Differential scanning fluorimetry**

For differential scanning fluorimetry (DSF), i.e. thermal shift assay and kinetic stability measurement, MPO_2_-, MPO_2_+L1-, MPO_5_- or pp65(417-426) peptide exchanged HLA-B7 monomers were used in a concentration of 2µM and analyzed on a StepOnePlus RT-PCR instrument (Applied Biosystems, Foster City, CA, USA), respectively. Samples were measured in a final volume of 20µl in MicroAmp^R^ Fast Optical 96-well reaction plates (Applied Biosystems) containing 150mM NaCl, 10mM HEPES (pH 7.4), 3mM EDTA and 10x SYPRO orange (Thermo Fisher Scientific) as described previously ([Hellman et al., 2016](#_ENREF_21)). Analysis was done following the recommendations described elsewhere ([Niesen et al., 2007](#_ENREF_43)) using the Microsoft Excel-based tool “DSF Analysis v3.0.2” available under the following link (<ftp://ftp.sgc.ox.ac.uk/pub/biophysics>). For accurate ﬁtting and calculation of V_50_-values, the Boltzmann equation was performed using GraphPad Prism (version 7.04, San Diego, USA). The kinetic stability was analyzed at constant 37°C and fluorescence was measured every 5 minutes. The fluorescent data were fit to a two-phase decay function and slowest rates for half-lives were calculated using GraphPad Prism (version 7.04, San Diego, USA).

**Purification and LC-MS/MS analysis of HLA-I peptides.**

The MPO_2_ and MPO_5_ ligands were identified using an immunopeptidomic approach as previously described ([Klar et al., 2014](#_ENREF_29)). For the targeted identification and relative quantification of both peptides, HLA-I peptides were purified from 5x10^8^ cells of each cell line as previously described ([Bassani-Sternberg et al., 2016](#_ENREF_6)). Heavy versions of the MPO_2_ and MPO_5_ peptides were synthetized using 13C6 15N leucine (Cambridge Isotope Laboratories, Inc.) and added to each sample at a 1 pmol/µl concentration. Peptides were loaded in buffer A (0.1% formic acid) on a 50cm long column, in house packed with ReproSil-Pur C18-AQ 1.9μm resin (Dr. Maisch HPLC GmbH), and eluted during a 90-minute linear gradient of 5-30% buffer B (80% ACN, 0.1% formic acid) on an EASY-nLC 1200 system coupled online to a Q Exactive HF-X mass spectrometer (Thermo Fisher Scientific). The mass spectrometer was operated in a data dependent mode, with full scans at resolution 60,000 and target value of 3e6 ions with a maximum injection time of 80ms. The top ten ions with charge 1-4 were accumulated to a target value of 1e5 for a maximum injection time of 120ms, fragmented by higher-energy collisional dissociation (HCD) and acquired with a resolution of 15,000. Selected Ions Monitoring (SIM) scans were added to record simultaneously the light and heavy couple of either the MPO_2_ or the MPO_5_ peptide. Each SIM scan was set with resolution 120,000, 1e5 ions accumulation target with a maximum injection time of 230ms, 1.4 m/z isolation window and 0.4 m/z isolation offset. For data analysis, chromatograms were processed with Skyline (version 3.7.0.11317).
